# Auxin and Jasmonic Acid Signalling Crosstalk Mediates Plant Growth Regulator-Stimulated Epigallocatechin Gallate Accumulation in Tea Plants (*Camellia sinensis*)

**DOI:** 10.3390/plants15182764

**Published:** 2026-09-09

**Authors:** Yunfei Hu, Lijia Liu, Xinyu Zeng, Xiaofeng Lu, Yanming Tuo, Yufang Wang, Zhirong Zhu, Qiufang Zhu, Yutao Shi, Liangyu Wu, Yue Zhang, Jinke Lin

**Affiliations:** 1 College of Horticulture/Anxi College of Tea Science, Fujian Agriculture and Forestry University, Fuzhou 350002, China; 2 Institute of Quality Standards & Testing Technology for Agro-Products, Fujian Academy of Agricultural Science, Fuzhou 350003, China; 3 College of Tea and Food Sciences, Wuyi University, Wuyishan 354300, China

**Keywords:** plant growth regulators, auxin, jasmonic acid, catechin, endogenous hormones

## Abstract

Epigallocatechin gallate (EGCG) imparts distinctive health benefits and flavor to tea, and its accumulation is modulated by plant growth regulators (PGRs). However, the regulatory mechanisms of Paclobutrazol (PAC) and Lovastatin (LS) underlying EGCG biosynthesis remain uncharacterized. We integrated quantitative analysis, transcriptomics, and metabolomics to investigate the regulatory effects of exogenous PAC and LS on EGCG accumulation in tea plants and systematically elucidate the underlying molecular mechanism governing EGCG biosynthesis. The results demonstrated that exogenous PAC and LS treatments significantly elevated the accumulation of EGCG, as well as endogenous auxin and jasmonic acid (JA) contents. The variation trend of these key metabolites was highly consistent with the accumulation patterns of upstream flavonoid components, including eriodictyol, delphinidin, and epigallocatechin. Transcriptomic profiling further verified the critical involvement of auxin and JA signal transduction pathways in PGR-induced EGCG differential accumulation, and we screened a total of 14 auxin-related and 8 JA-related core signal regulatory factors. Furthermore, integrated bioinformatic analyses and antisense oligonucleotide (AsODN) functional validation experiments revealed that the core hormone signaling genes *CsARF2* and *CsMYC2* regulate the expression of *CsSCPL16* through the transcription factor *CsMYB80*, and ultimately promote the conversion of epigallocatechin to EGCG and promote the accumulation of EGCG in tea plants. Collectively, the PGRs boost EGCG biosynthesis by mediating endogenous auxin and JA signal transduction. These results support the development of targeted agronomic practices to improve tea quality and lay a theoretical basis for expanding the industrial exploitation of tea bioactive constituents.

## 1. Introduction

Tea is one of the most popular non-alcoholic beverages, processed from the tender shoots of *Camellia sinensis* (L.) O. Kuntze, owing to its unique flavor and the health benefits [1]. EGCG is the core component of sensory quality and health care quality, which has the effects of anti-cancer, anti-hypertension, and anti-diabetes [2,3,4]. The biosynthesis of EGCG is affected by different genetic characteristics of tea plants, stress response, biological stress, and climate response [5,6,7,8]. Phytohormones assume a pivotal role in orchestrating plants’ secondary metabolism, deftly coordinating intricate signaling networks [9]. Studies have shown that the promoters of *CsPAL*, *CsCHS*, *CsANR*, *CsF3H*, *CsDFR* and other structural genes in the EGCG biosynthesis pathway of tea plants contain the response elements of salicylic acid (SA), gibberellin (GA), jasmonic acid (JA), cytokinin (CTK), auxin and abscisic acid (ABA) [8]. Exogenous hormones such as auxin, JA, methyl salicylate, and brassinosteroid (BR) could accelerate the increase of EGCG content in tea plants [7,10,11,12]. There may be a correlation between plant hormones, indicating that crosstalk between hormones may play a key role in flavonoid biosynthesis [13,14,15,16].

Paclobutrazol (PAC) and Lovastatin (LS) are important Plant growth regulators (PGRs), which play an important role in regulating plant hormones such as auxin, JA, and GA in plants. PAC can reduce the content of endogenous GA and auxin in plants and increase the content of ABA [17,18]. LS can increase the content of endogenous auxin, ABA, BR, GA, and JA in plants, thus affecting plant growth and metabolism [19]. PAC promoted plant growth of *Pinus koraiensis* (branch diameter, shoot and lateral branch elongation, above and below ground biomass and second order root quantity), strengthened antioxidant capacity and lignin deposition (increased SOD, POD activities and lignin content; decreased relative conductivity and malondialdehyde), and improved photosynthetic capacity with higher net photosynthetic rate, stomatal conductance, transpiration rate and maximum net photosynthetic rate [20,21]. Meanwhile, it reprograms secondary metabolic pathways to cope with unfavorable environmental stresses. In walnut (*Juglans regia*), PAC elevated IAA levels and the abundance of osmotic adjustment compounds such as proline and glutathione, while reducing chlorophyll, soluble sugars, and starch, thereby retarding plant growth [22]. Under salinity stress conditions, PAC treatment significantly induced the expression of flavonoid biosynthesis-related genes (*MYB111* and *MYB12*), markedly enhanced anthocyanin accumulation and vegetative growth, and alleviated the adverse effects of salinity stress in *Brassica napus* [23]. PAC significantly increased the total flavonoid content in flower buds [24,25], enhanced fruit coloration and anthocyanin accumulation [26], and induced the production of phenolic compounds [27]. The appropriate concentration of PAC treatment increased the activity of PAL and 4CL in maize [28], and affected the expression of PAL, ANS, CHS, and F3H in lily leaves, thereby inducing an increase in flavonoid content [29].

LS inhibits the cytosolic mevalonate (MVA) isoprenoid pathway, and this inhibitory effect can be rescued by exogenous mevalonic acid in *Arabidopsis thaliana*. Application of LS to methylerythritol phosphate (MEP) pathway-deficient cla11 mutants causes simultaneous blockage of the MVA and MEP pathways, leading to complete chlorophyll loss and plastid structural damage [30]. In soybean seedlings, LS represses the MVA pathway in a dose-dependent manner, impairs root meristem activity, inhibits primary and lateral root growth, and reduces sterol content, together with downregulated *GmHMGR* expression; consistently, *Arabidopsis* overexpressing *GmHMGR4* or *GmHMGR6* exhibits longer primary roots, higher levels of total sterol, squalene, and the MEP-derived end product tocopherol [31]. In apple stem cuttings, LS triggers moderate adventitious root formation by elevating indole-3-acetic acid, decreasing zeatin riboside, repressing cytokinin-related genes (*MdARR3*, *MdARR5*, *MdARR5-2*, *MdAHK4*, *MdCKX5*) and inducing auxin signalling genes (*MdIAA23*, *MdARF7*, *MdARF19*) [32]. When applied to decapitated apple seedlings to block local CTK production, LS largely abolishes decapitation-induced axillary bud outgrowth, decreases zeatin riboside levels in buds and internodes, suppresses early upregulation of bud activation related genes including type A-ARRs, auxin transport and axillary meristem identity genes, and further inhibits cell cycle related gene activation [33]. For Astragalus mongholicus root cultures, LS suppresses 3-hydroxy-3-methylglutaryl-CoA reductase (HMGR) activity and moderately increases triterpenoid accumulation, and combined treatment with cis-3-hexenal produces synergistic effects to greatly raise astragaloside and cycloastragenol contents [34]. LS dose-dependently suppresses in vitro tuberization of Solanum tuberosum stolons, delays tuber initiation, reduces tuber fresh weight and tuberization rate, and induces abnormal tuber morphology; it reduces bioactive free base CTK and disrupts hormone homeostasis with increased ABA, JAs and SA as well as lowered ratios of active cytokinins to GA_3_, ABA and JAs [35]. In wheat, LS downregulates *TaNRT1.1*, *TaNRT1.3* and *TaNRT1.4*, represses nitrogen metabolism, reduces total leaf nitrogen content and the activities of key nitrogen assimilating enzymes NR, NiR and GS, and ultimately induces leaf senescence [36].

At present, advances have been made in dissecting the mechanisms underlying EGCG biosynthesis in tea plants, as well as the regulatory effects of exogenous growth regulators on flavonoid metabolism. However, few studies have specifically focused on the modulation of EGCG biosynthesis by PGRs based on PAC and LS, which has limited the rational development and application of such regulators in tea cultivation. To explore the interactive effects and regulatory mechanisms of PAC and LS on EGCG accumulation in tea plants, four treatments, including Control (Ct), PAC, LS, and combined PAC-LS, were established under field conditions in this study. UPLC and LC-MS were utilized to determine the transcript levels and metabolite abundances of the EGCG biosynthetic pathway. This study aimed to elucidate the intrinsic molecular mechanisms by which PAC and LS promote EGCG accumulation in tea plants, providing a feasible agronomic strategy for improving the accumulation of tea bioactive compounds.

## 2. Materials and Methods

### 2.1. Plant Materials

Ten-year-old tea plants (*Camellia sinensis* (L.) O. Kuntze cv. ‘Huangdan’) with consistent growth and no pests or diseases were selected in April 2023 from the cultivated tea garden located in Meizhuang Village, Anxi County, Fujian Province, China (latitude: N 24°54′, E 117°51′), which belongs to the scientific research experimental tea garden of Fujian Agriculture and Forestry University, and is a commonly cultivated tea crop and not listed in CITES. All plant materials were obtained from cultivated resources and used in compliance with institutional, national, and international guidelines and regulations. According to previous research, 50 μmol·L^−1^ PAC [37] and 100 μmol·L^−1^ LS [38] were selected, and PAC and LS were uniformly dissolved in pure water. Before spraying, Tween-20 at a concentration of 0.1% was added to each group and sprayed on the new shoots of tea trees, and a combination of 50 μmol·L^−1^ PAC and 100 μmol·L^−1^ LS (P-L) was sprayed until runoff occurred. PAC and LS were obtained from Shanghai Yuanye Bio-Technology Co., Ltd. (Shanghai, China), both with purities above 95%. Distilled water was applied as the Ct). The experimental plots were arranged in a completely randomized block design, with each plot containing 12 tea plants and covering an area of 5 m^2^. Each treatment was replicated three times. The experiment was conducted on a sunny day with temperatures ranging from 15 °C to 25 °C. At 24 h after spraying, tea shoots consisting of one apical bud and two leaves were collected, immediately frozen in liquid nitrogen, and stored at −80 °C (DW86L626, Haier, Qingdao, China) for subsequent experiments.

### 2.2. Determination of Catechin Monomers Content

The content of catechin monomers was determined using a method described by Xiang et al. [39], with slight modifications to accommodate the experimental conditions. Briefly, tea powder samples were extracted with 70% methanol, and the resulting extracts were analyzed by ultra-performance liquid chromatography (UPLC) using a Waters Acquity UPLC HSS T3 column. The column temperature was maintained at 35 °C, the flow rate was set at 1.0 mL·min^−1^, and the detection wavelength was 278 nm. The mobile phase A consisted of 97% acetonitrile, 2% acetic acid, and 89% ultrapure water, while mobile phase B was composed of 80% acetonitrile and 20% ultrapure water. Quantification was performed using external calibration with standard catechin compounds. During sample analysis, a blank solvent was injected every six samples to monitor potential contamination or carryover.

### 2.3. Hormone Detection

Hormone quantification was performed by MetWare Biotechnology Co., Ltd. (Wuhan, China) based on the AB Sciex QTRAP 6500 LC-MS/MS platform. Quantitative determination of six endogenous hormones, including ABA, auxin, CTK, JA, GA, and SA, was conducted according to the Li et al. method [40]. Full names of the specific internal-standard compounds for hormone quantification are provided in Supplementary Table S1. In Brief, precisely weighed 50 mg tissue samples were snap-frozen with liquid nitrogen, then homogenized and extracted in 1 mL mixed solvent (methanol:formic acid:ultrapure water = 15:1:4, *v*/*v*/*v*). Prior to extraction, 10 μL internal standard cocktail (100 ng·mL^−1^) was supplemented into each extract to serve as the quantification reference. The mixture underwent vigorous vortexing for 10 min followed by a 5 min centrifugation step. The collected supernatant was vacuum-dried, reconstituted with 100 μL 80% (*v*/*v*) methanol, and passed through a 0.22 μm filter membrane before injection into the UPLC-ESI-MS/MS platform for subsequent metabolite profiling.

### 2.4. Metabolomic Determination

The quantification of widely targeted metabolites was conducted by a commercial service provider (MetWare Biotechnology, Wuhan, China) in accordance with a previously reported method [41]. The samples were ground into powder using a vacuum freeze-drying technique (30 Hz, 1.5 min). A 50 mg aliquot of each sample was mixed with 1200 μL of 70% methanol aqueous solution at 20 °C, followed by vortexing and centrifugation at 13,523× *g* (12,000 rpm) for 3 min. The supernatant was filtered through a 0.22 μm filter membrane, which was analyzed using a UPLC-ESI-MS/MS system (UPLC: ExionLC™AD, https://sciex.com.cn/). Differentially accumulated metabolites (DAMs) were screened using the thresholds of VIP ≥ 1 and ∣log2FC∣ ≥ 1.

### 2.5. Transcriptomic Analysis of Tea Samples

Total RNA was isolated from tea apical buds and two attached leaves sampled in April 2023 using TRIzol reagent, with three biological replicates. RNA libraries were sequenced on Illumina Seq 6000, and raw data were processed as in Wang et al. [42]. Clean reads were mapped to the ‘Huangdan’ tea reference genome via HISAT v2.1.0. DESeq2 was used to screen differential expressed genes (DEGs) with criteria |Log_2_FC| ≥ 0.5 and *p* < 0.05 [43]. KEGG database was used for gene function and metabolic pathway enrichment analysis.

### 2.6. Validation of Quantitative Real-Time PCR (qRT-PCR)

RNA from transcriptome samples was reverse-transcribed into first-strand cDNA. Gene-specific primers were designed via Primer3Plus https://www.bioinformatics.nl/cgi-bin/primer3plus/primer3plus.cgi/ (accessed on 15 July 2025), with all sequences listed in Supplementary Table S2. qRT-PCR was run on a qTower3G (Jena, Germany) instrument under standard thermal cycles: 30 s at 95 °C, then 40 cycles of 5 s at 95 °C and 36 s at 60 °C. β-actin served as the internal reference, and relative gene expression was calculated by the 2^−^^ΔΔCt^ method. This qRT-PCR assay verified DEG expression trends and validated the reliability of RNA-seq data.

### 2.7. Antisense Oligonucleotide (AsODN) Experiments

Sense oligodeoxynucleotides (sODN) and AsODN primer sequences of *CsARF2* (HD. 14G0019970), *CsMYC2* (HD. 07G0018360), and *CsMYB80* (HD. 08G0030520) were designed and screened via the Soligo web server (https://sfold.wadsworth.org/cgi-bin/soligo.pl (accessed on 18 May 2026)). Antisense sequences of the screened primers (Supplementary Table S3) were submitted to the Tea Plant Information Archive 2.0 (TPIA 2.0) BLAST platform (https://tpia.teaplants.cn/Blast.html (accessed on 25 May 2026)) to retrieve target gene CDS regions for gene silencing. Synthesized sODN and AsODN powders were diluted with ultrapure water to a working concentration of 50 μmol·L^−1^ with a previously reported method [44]. Tea apical buds and two leaves collected from ‘Huangdan’ were soaked in centrifuge tubes filled with the above oligonucleotide solutions. After 24 h incubation, all treated samples were wrapped in aluminum foil, snap-frozen rapidly in liquid nitrogen, and preserved at −80 °C for later gene expression analysis of *CsARF2* (HD. 14G0019970), *CsMYC2* (HD. 07G0018360), *CsMYB80* (HD. 08G0030520), *CsSCPL16* (HD. 1307844), and EGCG detection.

### 2.8. Data Analysis

Excel 2019 was used for data processing. IBM SPSS 25.0 was employed for variance analysis and Pearson correlation analysis. *t*-test was used for two-group comparisons, and Duncan’s test was applied to identify significant differences among multiple groups. KEGG and GO enrichment analyses were performed using the Metabolic Cloud Platform (https://cloud.metware.cn/), and histograms were plotted with GraphPad Prism 9. Transcription factors were predicted via PlantTFDB (https://planttfdb.gao-lab.org/), while transcription factor binding sites were predicted using TBtools-II v2.096.

## 3. Result

### 3.1. PAC and LS Induced Accumulation of EGCG, Endogenous Auxins and JAs

**Figure 1:**
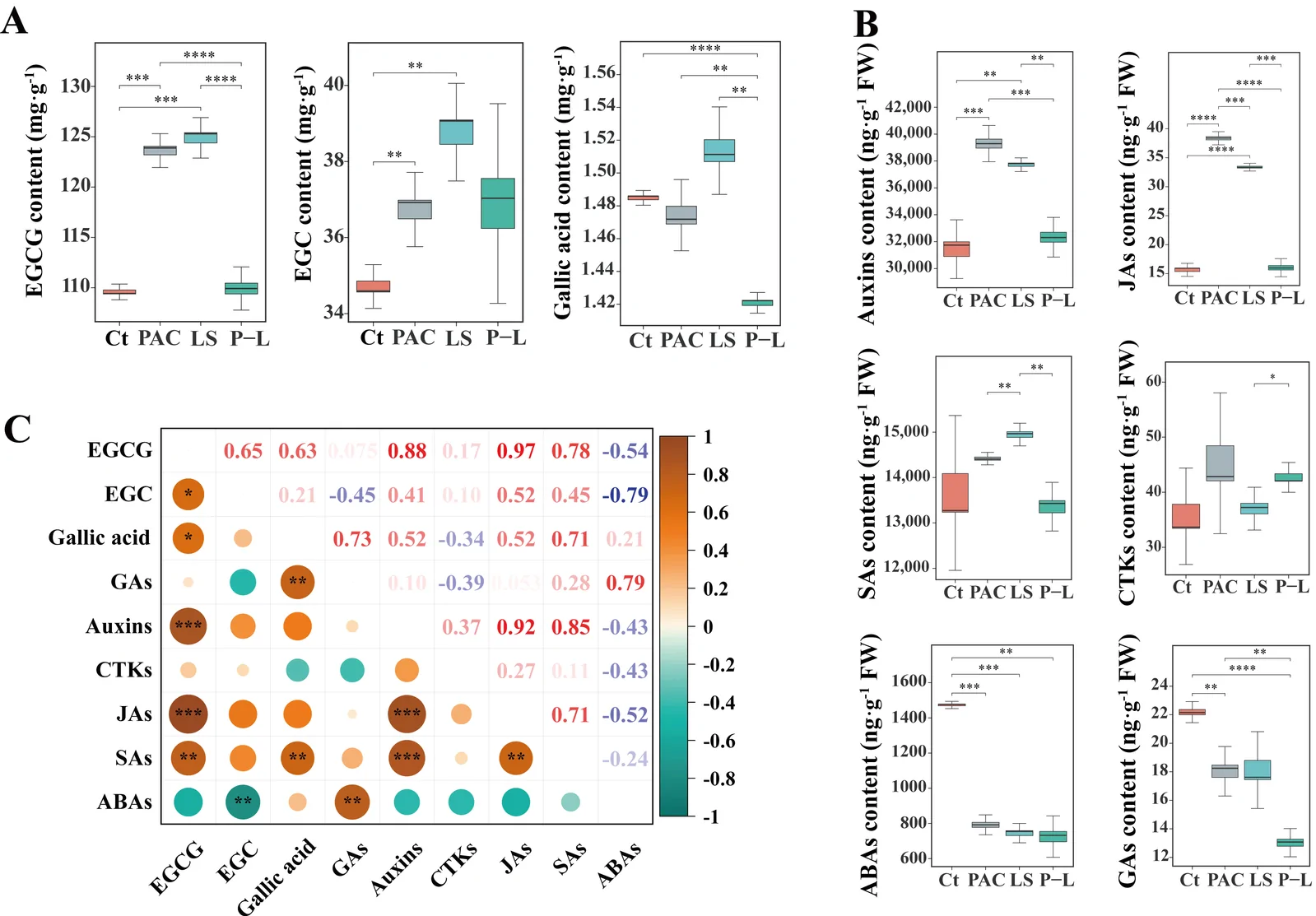
Relationship between EGCG and endogenous hormones in tea shoots. (**A**) The content of EGCG, EGC, and gallic acid in tea shoots; (**B**) The content of endogenous hormones in tea shoots; Supplementary Table S4 contains various hormone components; (**C**) The correlation between EGCG, EGC, gallic acid, and endogenous hormones. The size of each dot represents the correlation coefficient. Significance levels: * *p* < 0.05, ** *p* < 0.01, *** *p* < 0.001, **** *p* < 0.0001.

Compared with the Ct group, EGCG contents treated with PAC or LS increased extremely significantly (*p* < 0.001), with growth rates of 12.68% and 13.80%, respectively. EGC contents were also extremely significantly elevated (*p* < 0.01), while no significant difference was observed in gallic acid contents. By contrast, under combined PAC-LS spraying treatment, no significant changes were detected in EGCG and EGC contents relative to Ct, whereas gallic acid contents decreased extremely significantly (*p* < 0.001) (Figure 1A). LC-MS/MS was employed to quantify a total of 48 endogenous phytohormone components in tea new shoots from different treatments, consisting of 6 GAs, 15 auxins, 16 CTKs, 2 JAs, 3 ABAs and 6 SAs (Supplementary Table S4). Compared with the Ct, the endogenous auxin and JA contents in tea shoots increased extremely significantly under PAC and LS treatments (*p* < 0.01), whereas the endogenous ABA and GA contents decreased extremely significantly (*p* < 0.01), and no significant differences were observed in SA and CTK contents (Figure 1B). Pearson correlation analysis revealed that auxins and JAs were significantly positively correlated with EGCG content (r > 0.88, *p* < 0.01) (Figure 1C). Collectively, treatments with PAC and LS significantly promoted the accumulation of EGCG and its precursor EGC in tea plants and simultaneously elevated the levels of endogenous auxins and JAs. However, combined foliar spraying of PAC and LS failed to trigger such alterations. Specifically, compared with the Ct, the contents of EGCG, auxin, and jasmonic acid showed no significant changes under the P-L treatment, and these values were markedly lower than those under individual PAC and LS spraying treatments. This is likely attributable to mutual antagonism generated by P-L co-application; the respective inductive effects of PAC and LS on auxin- and JA-dependent EGCG biosynthesis are offset, and consequently EGCG accumulation reverts to a level close to that of the Ct. Nevertheless, this phenomenon and its underlying regulatory mechanism merit further exploration in future studies.

### 3.2. PAC and LS Influenced Flavonoid Metabolic Profiles to Enhance EGCG Accumulation

**Figure 2:**
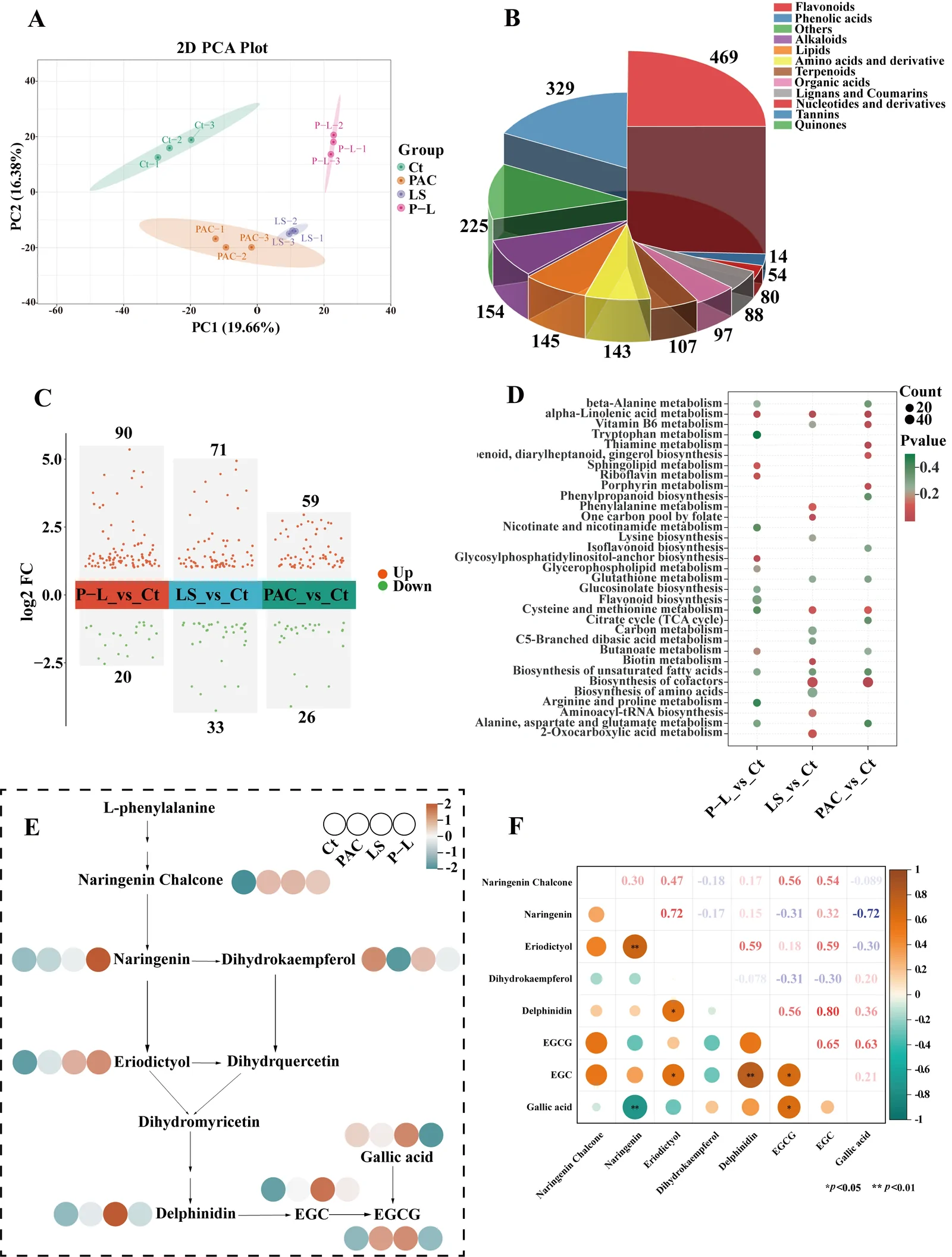
Metabolomic analysis of tea shoots after different treatments. (**A**) PCA of metabolites under different treatments. (**B**) Classification of metabolites in different treatment groups. (**C**) Number of differential metabolites. (**D**) KEGG enrichment analysis of DAMs. (**E**) Differential metabolites of EGCG metabolic pathway. (**F**) Correlation between EGCG and differential metabolites. The size of each dot represents the correlation coefficient.

To profile metabolite changes under treatments, we performed widely-targeted metabolomics analysis. A total of 1885 metabolites were identified. The four groups of samples were well separated, and the three replicates in each treatment group were relatively concentrated (Figure 2A). Significant differences in metabolites were observed among the different treatment groups (Figure S1). The main metabolites included 469 flavonoids, 329 phenolic acids, 154 alkaloids, 145 lipids, 143 amino acids, and their derivatives (Figure 2B). Compared with the Ct, PAC and LS induced differential accumulation of 104 and 85 metabolites, respectively, indicating that these metabolites are key compounds changing synchronously with EGCG. (Figure 2C). KEGG enrichment analysis revealed that DAMs were significantly enriched in flavonoid biosynthesis, phenylalanine metabolism, tryptophan metabolism, and other related pathways (Figure 2D). Seven DAMs participating in EGCG biosynthesis were further identified, including naringenin chalcone, naringenin, dihydrokaempferol, eriodictyol, delphinidin, EGC, and gallic acid. (Figure 2E). Correlation analysis with EGCG content showed that EGC and gallic acid, as precursors of EGCG biosynthesis, were significantly positively correlated with EGCG content. EGC was significantly positively correlated with delphinidin and eriodictyol, while naringenin was significantly negatively correlated with gallic acid (Figure 2F). Therefore, PAC and LS may induce eriodictyol and delphinidin in the flavonoid pathway to regulate EGC content, which in turn affects EGCG content.

### 3.3. PAC and LS Modulated CsSCPL Expression to Alter EGCG Biosynthesis via CsMYB80

To evaluate the sequencing quality of transcriptome data, we summarized the clean reads obtained from RNA-seq. The number of clean reads per sample ranged from 46,304,574 to 51,798,202, and the overall sequencing error rate of the samples was less than 0.03% (Q20 > 97%, Q30 > 92%), indicating high overall sequencing accuracy (Supplementary Table S5). The Clean Reads were aligned with the genome of tea plant variety ‘Huangdan’, and the average alignment rate was 95.07% (94.90–95.66%). The expression patterns obtained by RT-qPCR were consistent with those from RNA-seq, confirming the reliability of transcriptome analysis (Figure S2). The four groups of samples were well separated, and the three biological replicates of each treatment group were relatively clustered. Compared with the Ct group, PAC treatment produced 2106 DEGs (802 upregulated, 1304 downregulated), while LS treatment yielded 2406 DEGs (992 upregulated, 1414 downregulated) (Figure S3), respectively, indicating their critical roles in regulating EGCG accumulation. Further KEGG enrichment analysis verified that plant hormone signal transduction, phenylpropanoid biosynthesis, and MAPK signaling pathway responded to PAC and LS treatments to modulate EGCG biosynthesis (Figure S4).

**Figure 3:**
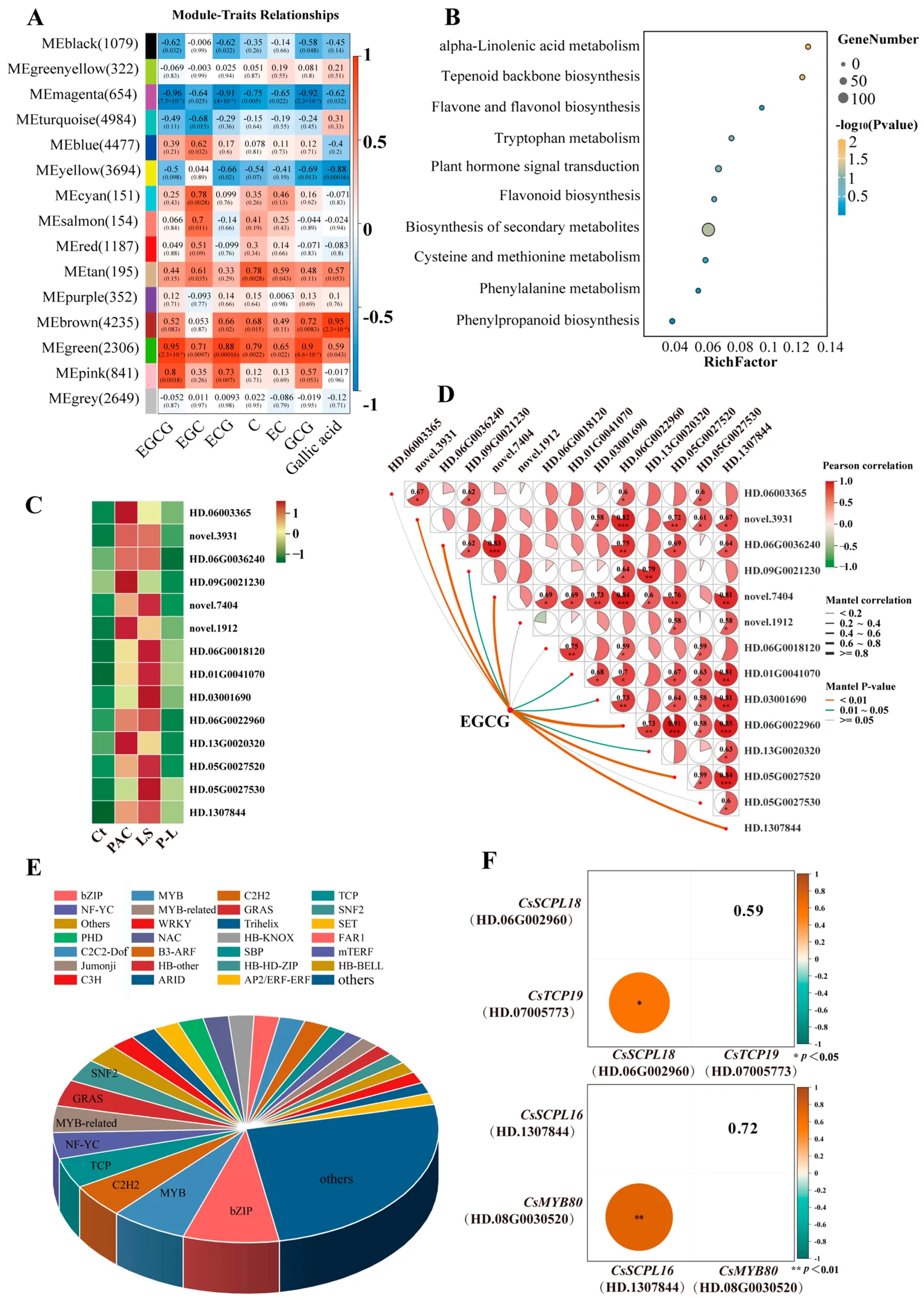
Weighted gene co-expression network analysis. (**A**) Correlations of catechin components and gallic acid content with gene expression modules. (**B**) KEGG enrichment analysis of genes in MEgreen module. (**C**) Differences in gene expression of EGCG synthesis pathway. (**D**) Correlation between EGCG and structural genes. (**E**) Transcription factor classification in MEgreen module. (**F**) Correlation between structural genes and transcription factors. Significance levels: * *p* < 0.05, ** *p* < 0.01, *** *p* < 0.001.

The MEgreen module exhibited a strong positive correlation with EGCG accumulation (r = 0.95, *p* < 0.01) (Figure 3A). Functional annotation of the 2306 genes within the MEgreen module was significantly enriched in several key metabolic pathways, including plant hormone signal transduction and flavonoid biosynthesis, flavone and flavonol biosynthesis (Figure 3B). Within the EGCG biosynthetic pathway, 14 structural genes were identified (Figure 3C). Notably, the expression levels of *CsDFR* (HD.06G0036240), *CsSCPL* (HD.06G0022960, HD.05G0027520, HD.1307844), and *CsANS* (HD.09G0021230) were significantly upregulated under PAC and LS treatments, and the expression of *CsDFR* and three *CsSCPL* were significantly positively correlated with EGCG content (r > 0.8, *p* < 0.01) (Figure 3D), indicating their potential roles as key regulators in EGCG biosynthesis induced by PAC and LS.

Transcription factors (TFs) are involved in EGCG biosynthesis in tea plants; analysis showed that transcription factor prediction analysis identified a total of 187 TFs in the MEgreen module (Figure 3E), and 39 differentially expressed transcription factors contained binding sites in the promoters of the four core structural genes involved in EGCG biosynthesis (Supplementary Table S6). Among these, the expression of *CsTCP19* (HD.07005773) was significantly positively correlated with *CsSCPL18* (HD.06G0022960) (r = 0.59, *p* < 0.05), while *CsMYB80* (HD.08G0030520) showed a strong positive correlation with *CsSCPL16* (HD.1307844) (r = 0.72, *p* < 0.01) (Figure 3F). Prediction of response elements showed that the promoter region of transcription factor *CsMYB80* contained both auxin response elements and JA response elements, while the promoter region of *CsTCP19* contained auxin response elements (Supplementary Table S7). These results suggest that *CsMYB80* may act as a potential transcriptional regulator in response to auxin and JA signals to regulate the expression of EGCG biosynthetic genes.

### 3.4. PAC- and LS-Triggered Auxin and JA Signals Regulated EGCG Biosynthesis Through CsARF2 and CsMYC2

**Figure 4:**
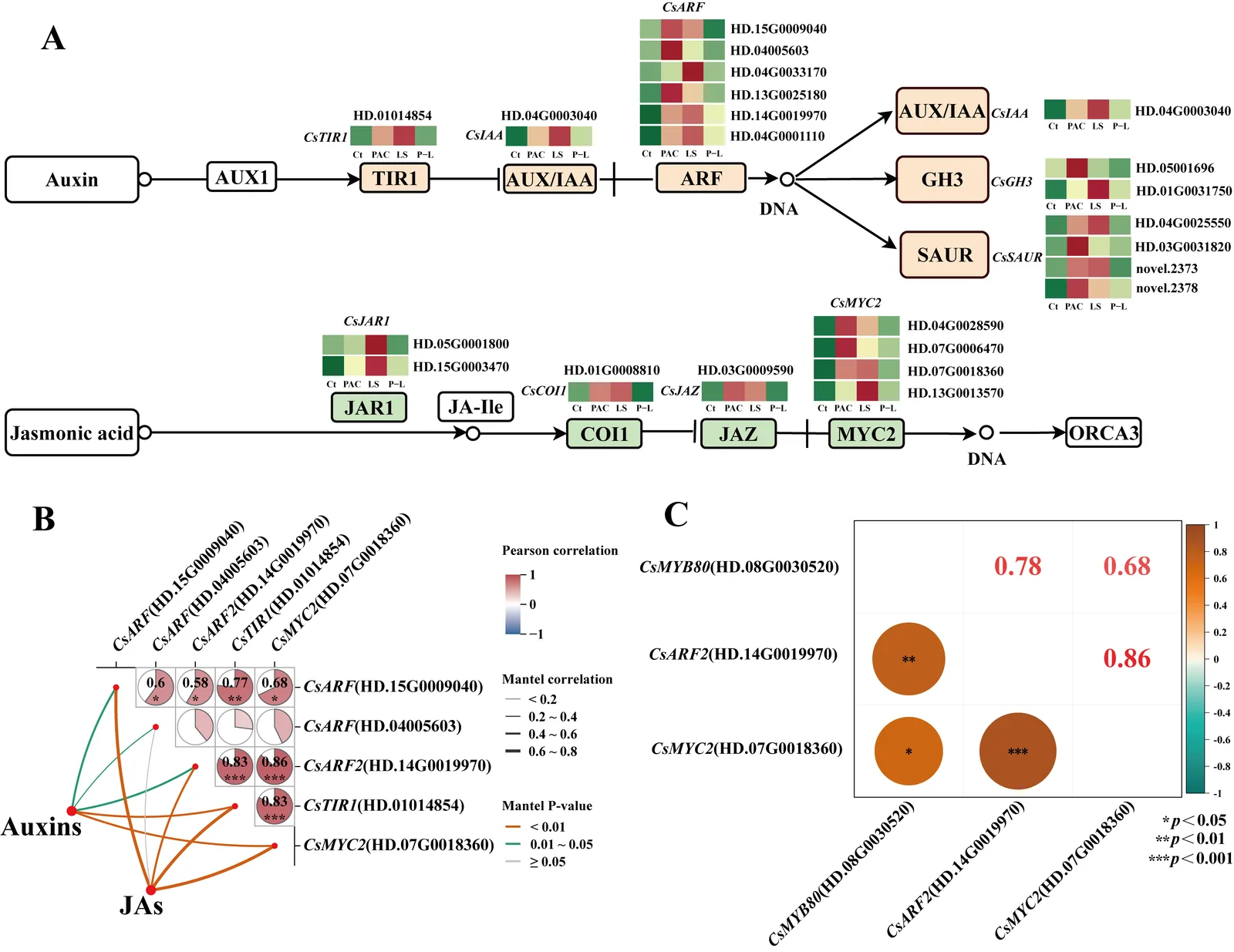
Analysis of endogenous auxin and JA signals in tea plants. (**A**) Differential signal transduction factors of auxin and JA pathways. (**B**) Correlation of endogenous auxins, JAs, and signal transduction factors, with different colors corresponding to Mantel *p*‑values.(**C**) Correlation of signal transduction factors and transcription factors. Significance levels: * *p* < 0.05, ** *p* < 0.01, *** *p* < 0.001.

This study revealed synchronous alterations in EGCG accumulation alongside endogenous auxin and JA levels, followed by identification of auxin and JA signal transduction factors governing EGCG biosynthesis. A total of 14 auxin and 8 JA signal transduction factors were screened in the MEGreen module (Figure 4A). Correlation analysis showed that *CsARF* (HD.15G0009040, HD.04G005603, HD.14G0019970) and *CsTIR1* (HD.01G014854) exhibited significantly positive correlations with auxin contents (r > 0.70, *p* < 0.01). Meanwhile, *CsMYC2* (HD.07G0018360) displayed a strong positive correlation with JA contents (r > 0.83, *p* < 0.01) (Figure 4B). Therefore, this study suggests that *CsARF*, *CsTIR1*, and *CsMYC2* respond to changes in auxin and JA contents, participate in signal transduction, and thereby regulate the biosynthesis of EGCG in tea shoots. Correlation analysis showed that *CsMYB80* was significantly positively correlated with *CsARF2* (HD.14G0019970) and *CsMYC2* (HD.07G0018360) (r ≥ 0.68, *p* < 0.05) (Figure 4C).

### 3.5. Functional Validation of CsARF2 and CsMYC2 in Regulating EGCG Biosynthesis via CsMYB80

**Figure 5:**
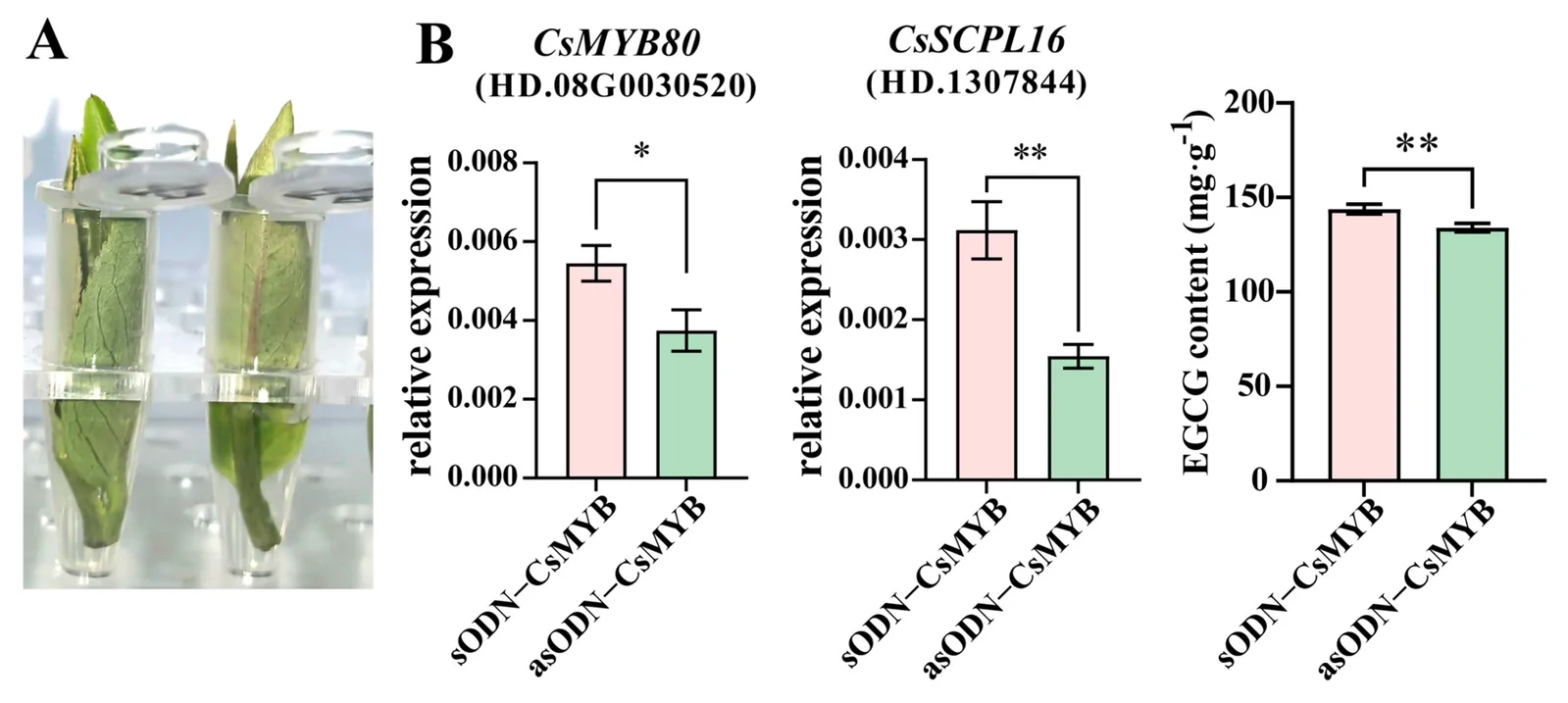

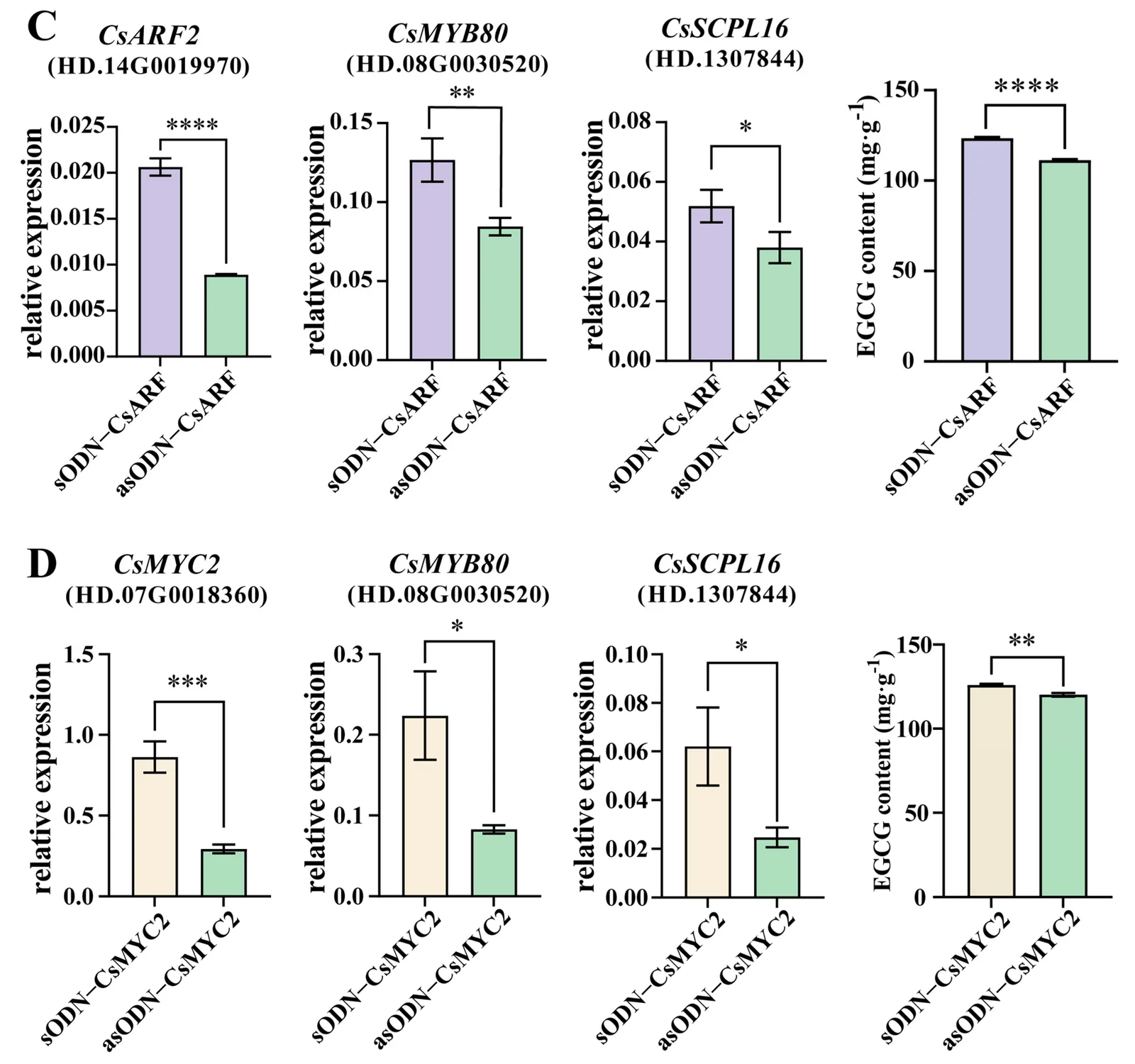
Silencing the expression of *CsMYB80, CsARF2,* and *CsMYC2* reduces EGCG content in tea shoots. (**A**) Tea shoots were immersed in AsODN and sODN solutions. (**B**) Antisense experiment significantly reduced the expression of *CsMYB80* and *CsSCPL16*. (**C**) Silencing *CsARF2* significantly reduced the expression of *CsMYB80* and *CsSCPL16*. (**D**) Silencing the expression of *CsMYC2* significantly reduced the expression of *CsMYB80* and *CsSCPL16*. Significance levels: * *p* < 0.05, ** *p* < 0.01, *** *p* < 0.001, **** *p* < 0.0001.

AsODNs were designed to individually silence *CsARF2*, *CsMYC2*, and *CsMYB80*, with corresponding sODNs set as the control. Tea shoots were immersed in AsODN and sODN solutions for 24 h (Figure 5A). Silencing of *CsMYB80* significantly reduced its transcript abundance by 31.24% (*p* < 0.05). Consistently, the expression level of its downstream gene *CsSCPL16* and EGCG content were markedly decreased by 50.54% and 6.84%, respectively (*p* < 0.01) (Figure 5B). Further silencing of *CsARF2* induced a 56.80% reduction in its transcript level (*p* < 0.0001); meanwhile, the expression of *CsMYB80* and *CsSCPL16* declined significantly, accompanied by a 9.79% drop in EGCG content (*p* < 0.0001) (Figure 5C). Similarly, silencing *CsMYC2* significantly suppressed the expression of its own gene, *CsMYB80*, and *CsSCPL16*, and reduced EGCG content (*p* < 0.01) (Figure 5D).

### 3.6. Potential Mechanism Underlying Synergistic Auxin and JA Signaling Governing EGCG Biosynthesis

**Figure 6:**
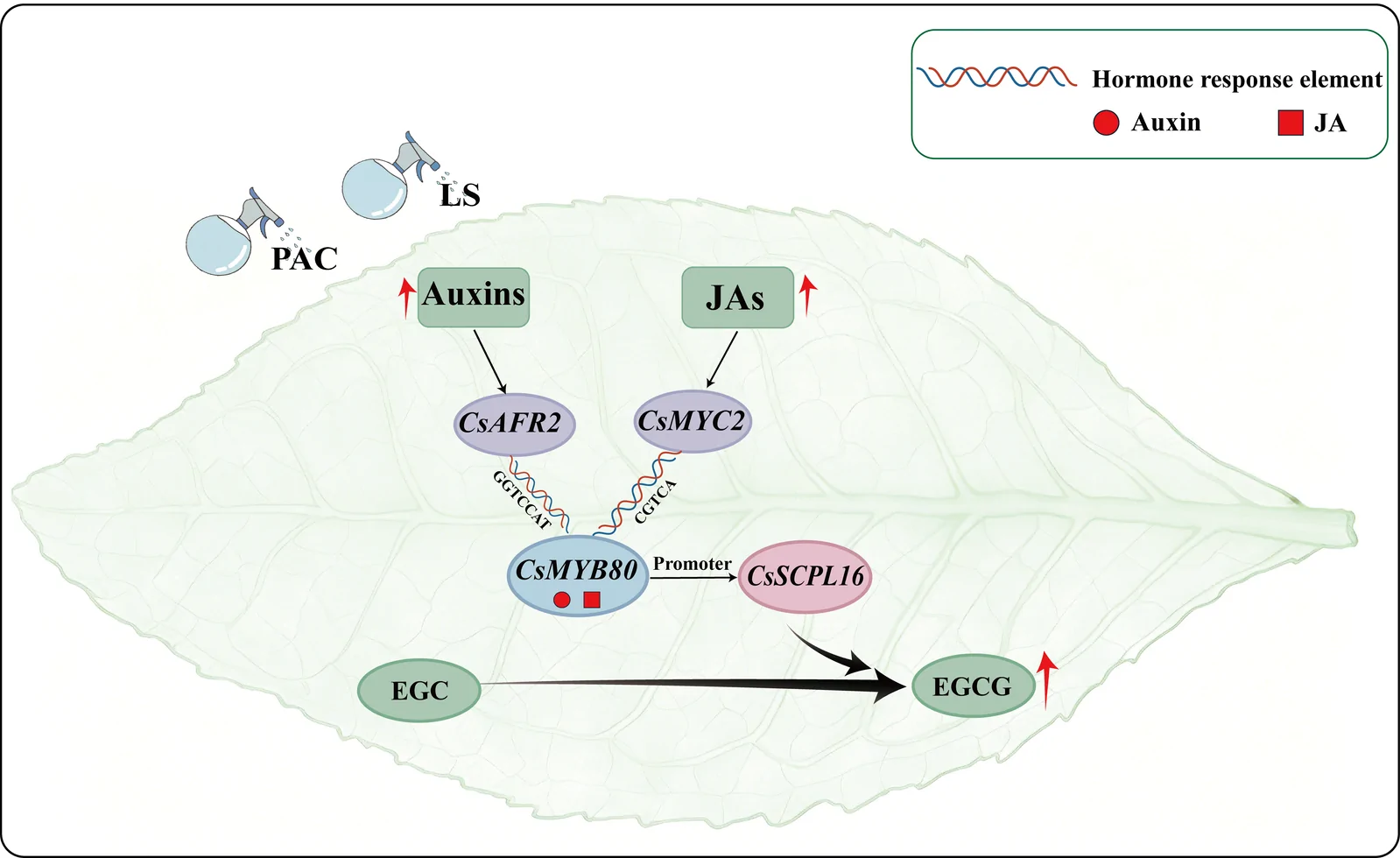
Potential mechanism map of auxin and JA signaling regulating EGCG in tea plants. The red arrow represents the increase in auxins, JAs, and EGCG content. The curly red and blue lines denote the hormone response element of *CsMYB80*.

The results suggested that the transcription factor *CsMYB80* (HD.08G0030520) may function as a central interactive node that integrates endogenous auxin and JA signaling in tea plants. Both the auxin-responsive transcription factor *CsARF2* (HD.14G0019970) and the JA-responsive transcription factor *CsMYC2* (HD.07G0018360) were verified to interact with *CsMYB80*. Based on these observations, we proposed a potential mechanism underlying the co-regulation of EGCG biosynthesis by endogenous auxin and JA. Under separate PAC and LS treatments, endogenous auxin and JA contents were significantly increased. The enhanced hormone signals further activated *CsARF2* and *CsMYC2*, which synergistically promote the expression of the potential key factor *CsMYB80*. Subsequently, the upregulated expression of the downstream structural gene *CsSCPL16* accelerated the catalytic conversion of EGC to EGCG, ultimately facilitating EGCG biosynthesis in tea plants (Figure 6).

## 4. Discussion

### 4.1. Exogenous PAC and LS Modulate Endogenous Hormone Levels to Remodel Flavonoid Biosynthesis and Enhance EGCG Accumulation

As typical PGRs, PAC and LS influence the accumulation of endogenous hormones and secondary metabolites in plants and play vital roles in promoting the biosynthesis of plant compounds and influencing other phytohormones [45,46]. In this study, the contents of endogenous hormones and EGCG in tea shoots changed under PAC and LS treatments, and the contents of auxins, JAs, and EGCG in tea leaves increased significantly, while the contents of GAs and ABAs decreased significantly. Meanwhile, auxins and JAs showed a significant positive correlation with EGCG content. Studies have confirmed that the contents of endogenous IAA, JA, and other hormones in grapes are positively correlated with flavonoid contents [47,48], which is consistent with the results of this study. Eriodictyol and delphinidin significantly affected the content of EGC. Moreover, EGC and gallic acid, key precursors of EGCG, showed significant positive correlations with EGCG. Previous studies have shown that EGCG content in tea plants under different ecological factors is consistent with the content of EGC, GCG, ECG, and chalcone [39]. Therefore, the accumulation of EGCG may be associated with alterations in endogenous auxin and JA contents, which promote the accumulation of eriodictyol and delphinidin, leading to an increase in EGC content and an accumulation of gallic acid in the shikimic acid pathway. These metabolic and hormonal changes collectively shape EGCG accumulation under PAC and LS treatments.

Notably, few published studies have reported the application of PAC and LS in plants. Individual PAC or LS treatment reprograms plant endogenous hormones, elevates auxin and JA levels, and promotes secondary metabolite accumulation [17,18,32,49]. However, mutual antagonism was observed under P-L combined treatment; We hypotheses that this antagonistic phenotype may result from pathway crosstalk and precursor competition within the isoprenoid pathway, given that PAC targets GA biosynthesis whereas lovastatin blocks the cytosolic MVA pathway. They each rely on endogenous auxin and JA to counteract the induction of EGCG biosynthesis. Nevertheless, these hypotheses remain to be verified by further experimental evidence.

### 4.2. Auxin and JA Signaling Factors Mediate the Biosynthesis of EGCG in Tea Plants

Phytohormones serve as core endogenous signals regulating plant growth and development, which modulate transcription factors and the expression of flavonoid biosynthetic genes [50]. Multiple prior studies have validated that exogenous IAA treatment elevates the expression of the CHS protein and boosts total flavonoid accumulation in tea plants [51]. Further research confirmed that IAA upregulates *CsCHS* and *CsANS* via *CsIAA* and *CsSAUR*, thereby raising endogenous EGCG levels [8]. Consistent with these published observations, our work found that auxin signal stimulation activated the auxin signaling regulator *CsARF2*, promoted the expression of *CsSCPL16*, and enhanced EGCG accumulation in tea plants.

JAs are well documented as pivotal regulators of flavonoid biosynthesis. Elevated JA levels trigger marked upregulation of transcription factor *CsMYC2*, which binds the promoters of *CsDFR-1*, *CsDFR-2*, *CsANR-1*, and *CsLAR* to activate their transcription and accelerate catechin production in tea [52]. Our data revealed that PAC and LS treatments also significantly boosted *CsMYC2* expression and confirmed that this factor targets *CsMYB80*, activating *CsSCPL16* transcription to raise cellular EGCG levels. JA governs the transcription of metabolic enzyme genes primarily via its master regulator *CsMYC2*, which reshapes secondary metabolite accumulation and coordinates plant growth [53]. Previous research also identified *CsMYC2* as a central mediator coordinating crosstalk between auxin and JA signaling cascades [54]. In summary, under PAC and LS stimuli, auxin and JA signals jointly upregulate *CsSCPL16* to elevate EGCG accumulation in tea plants, with *CsARF2* and *CsMYC2* serving as core signal mediators.

### 4.3. CsMYB80 Mediated Synergistic Hormone Signaling Regulating EGCG Biosynthesis in Tea Plants

Plant hormones exhibit complex interactions, including synergistic and antagonistic effects, in the regulation of metabolic pathways [55]. Previous studies have demonstrated that JA promotes auxin biosynthesis by upregulating the expression of auxin biosynthetic genes in plants [56], while exogenous auxin application can also significantly increase JA content [57]. Similarly, in our study, changes in endogenous auxin and JA contents in the tea plant were consistent and showed a significant positive correlation under stimulation by PGRs. Moreover, MYB proteins are known to regulate a wide range of plant processes, including primary and secondary metabolism, responses to biotic and abiotic stresses, and development [58]. In this study, *CsMYB80* was found to respond to the auxin signaling factor *CsARF2* and the JA signaling factor *CsMYC2*. This result is consistent with the findings of Uji et al. [59], who reported that MYB transcription factors can synergistically integrate auxin and JA signal transduction during plant defense responses. This experiment discovered three differentially expressed genes, including *CsSCPL*, and previous studies have confirmed that different *CsSCPL* genes have different functions, such as *CsSCPL4* containing the conserved catalytic triad S-D-H, and *CsSCPL5* having the alternative triad T-D-Y. *CsSCPL4* is a catalytic acyltransferase, while *CsSCPL5* is a non-catalytic companion paralog (NCCP) [60]. Furthermore, *CsMYB80* was found to bind to the promoter of *CsSCPL16* and promote its expression, thereby increasing EGCG content in tea plants. Li et al. also demonstrated a protein–gene interaction mechanism between *CsMYB* and *CsSCPL* [61]. Several studies have reported that MYB transcription factors such as *CsMYB34*, *CsMYB196*, and *CsMYB219* can modulate *CsSCPL4* expression to catalyze the conversion of EGC to EGCG [62,63]. In summary, the auxin signal factor *CsARF2* and the JA signal factor *CsMYC2* jointly mediate the upregulation of the potential key factor *CsMYB80*, which may bind to the structural gene *CsSCPL16* and promote expression, ultimately facilitating the conversion of EGC to EGCG. However, the precise molecular mechanism by which *CsMYB80* coordinates auxin and JA signal transduction to regulate EGCG biosynthesis remains to be further elucidated. Subsequent studies can systematically dissect its regulatory mechanism using gene overexpression, dual-luciferase reporter assays, and protein-protein interaction experiments to verify the synergistic regulatory relationship.

## 5. Conclusions

In this study, exogenous PAC and LS treatments significantly increased endogenous auxin and JA levels in tea leaves, accompanied by elevated levels of key flavonoid intermediates and EGCG. Our results demonstrate that auxin and JA signaling pathways act synergistically to promote EGCG biosynthesis. This regulatory process is mediated by hormone-responsive transcription factors that transcriptionally regulate the core structural genes involved in catechin galloylation. Integrated multi-omics analyses further reveal that *CsMYB80* functions as a potential core regulatory hub that bridges the auxin signaling module *CsARF2* and the JA signaling module *CsMYC2* to EGCG biosynthesis. Overall, this study offers mechanistic insights into plant growth regulator-dependent regulation of endogenous hormones underlying EGCG accumulation from both metabolic and molecular perspectives. These findings establish a theoretical basis for improving the functional quality of tea raw materials through endogenous hormone regulation and offer practical guidance for optimizing field agronomic management to enhance tea quality.

## Data Availability

The raw data of RNA-Seq are available in the SRA database under project accession PRJNA1430416 https://www.ncbi.nlm.nih.gov/sra/PRJNA1430416 (accessed on 1 April 2026). The other datasets during the current study are also available from the corresponding author upon request.

## Supplementary Materials

The following supporting information can be downloaded at: https://www.mdpi.com/article/10.3390/plants15182764/s1. Table S1. Full names of the specific internal-standard compounds for hormone quantification. Table S2. Primer sequence for qRT-PCR. Table S3. Design of AsODNs sequences for *CsARF2*, *CsMYC2*, and *CsMYB80*. Table S4. Contents of endogenous phytohormone components in tea shoots of different treatment groups (ng·g^−1^ FW). Table S5. Quality evaluation of RNA-seq data. Table S6. Transcription factor binding site of 4 structure genes. Table S7. Prediction of hormone responsive elements in transcription factors. Figure S1. Cluster heatmap of all detected metabolites in tea shoots under different treatments. Figure S2. FPKM value and qRT-PCR relative expression of differentially expressed genes. The histogram represents the FPKM value of the differentially expressed genes, and the line chart represents the relative expression of qRT-PCR of the differentially expressed genes. Different lowercase letters represent significant differences in gene expression detected by qRT-PCR at the *p* < 0.05 level. Figure S3. Transcriptome analysis of tea shoots. (A) PCA diagram of the overall sample. (B) Number of differentially expressed genes. Figure S4. KEGG enrichment bubble diagram of differential genes in different treatment groups. (A) Enrichment analysis of PAC vs. Ct. (B) Enrichment analysis of LS vs. Ct. (C) Enrichment analysis of P-L vs. Ct.

## Abbreviations

Plant growth regulator (PGR); Paclobutrazol (PAC); Lovastatin (LS); Epigallocatechin (EGC); Epicatechin gallate (ECG); Epigallocatechin gallate (EGCG); Gallocatechin acid (GCG); Jasmonic acid (JA); Abscisic acid (ABA); Gibberellic acid (GA); Salicylic acid (SA); Cytokinin (CTK).
