# Supplementary material for: Auxin and Jasmonic Acid Signalling Crosstalk Mediates Plant Growth Regulator-Stimulated Epigallocatechin Gallate Accumulation in Tea Plants (Camellia sinensis)
Source: Plants (Basel). 2026 Sep 9;15(18):2764. doi: 10.3390/plants15182764 (PMC13610577; doi:10.3390/plants15182764)
Supplement: Supplementary file 1 [file plants-15-02764-s001.zip › plants-4516489-supplementary.pdf]

**Supplementary Table S1 Full names of the specific internal−standard compounds for hormone quantification**

| substance | Corresponding internal standard information | | |
|---|---|---|---|
| ABA | d6−ABA | Czech | 034 2721 |
| ABA−GE | d6−ABA | Czech | 034 2721 |
| ABA−ald | d6−ABA | Czech | 034 2721 |
| IAA−Glu | d5−IAA−Val | Czech | 0311641 |
| IAN | [2H4]INDOLE−3−ACETONITRILE | Czech | 031 1852 |
| IAA−Val−Me | d5−IAA−Val | Czech | 0311641 |
| OxIAA | d2−IAA | isoreag | R−30387−100 mg |
| IAA−Asp | [2H5]INDOLE−3−ACETYL−L−[15N] ASPARTIC ACID | Czech | 031 1593 |
| IAA−Val | d5−IAA−Val | Czech | 0311641 |
| IAM | [2H5]−IAM | IsoReag | IR−21912 |
| TRA | Tryptamine−α,α,β,β−d4 HCl | CDN | D1546 |
| IAA−Ala | [2H5]INDOLE−3−ACETYL−L−[15N] ALANINE | Czech | 031 1583 |
| TRP | [2H5]−L−TRP | sigma | 615862−500 mg |
| IAA−Phe | d5−IAA−Val | Czech | 0311641 |
| ICA | Indole−3−carboxylic Acid−d5 | isoreag | IR−73457−25 mg |
| IAA−Glc | d2−IAA | isoreag | R−30387−100 mg |
| IAA | d2−IAA | isoreag | R−30387−100 mg |
| ICAld | Indole−3−carboxaldehyde−13C | TRC | 1614992 |
| MEIAA | d5−MEIAA | CDN | D−6165−10 mg |
| Indole | d2−IAA | isoreag | R−30387−100 mg |
| mT | d7−BAP | Czech | 030 0221 |

| | | | |
|---|---|---|---|
| K | d7−BAP | Czech | 030 0221 |
| iP7G | d6−iP9G | Czech | 030 0231 |
| tZOG | [2H5]trans−ZEATIN−O−GLUCOSIDE | Czech | 030 5121 |
| DHZROG | [2H7]DIHYDROZEATIN−O−GLUCOSIDE RIBOSIDE | Czech | 030 0652 |
| cZROG | 15N4−cZ | Czech | 0300321 |
| mT9G | d6−iP9G | Czech | 030 0231 |
| BAP9G | d6−iP9G | Czech | 030 0231 |
| 2MeSiP | d6−IP | Czech | 030 0161 |
| 2MeScZR | [2H5]2−METHYLTHIO−trans−ZEATIN RIBOSIDE | Czech | 030 5181 |
| DHZ7G | d7−DHZOG | Czech | 030 0642 |
| IP | d6−IP | Czech | 030 0161 |
| IPR | d6−IPR | Czech | 030 0171 |
| tZ | d5−tZ | IsoReag | IR−35064 |
| tZR | d5−tZ | IsoReag | IR−35064 |
| iPRMP | d6−iP9G | Czech | 030 0231 |
| tZ9G | [2H5]trans−ZEATIN−O−GLUCOSIDE | Czech | 030 5121 |
| tZRMP | d6−iP9G | Czech | 030 0231 |
| GA20 | [2H2]GIBBERELLIN A20 | Czech | 0322483 |
| GA3 | d2−GA4 | Czech | 0322531 |
| GA4 | d2−GA4 | Czech | 0322531 |
| GA7 | d2−GA4 | Czech | 0322531 |
| GA9 | d2−GA9 | Czech | 0322661 |
| GA8 | d2−GA4 | Czech | 0322531 |
| GA5 | d2−GA4 | Czech | 0322531 |
| JA−Phe | d5−JA | SCRSTANDARD | D−6936 |
| JA−Val | d5−JA | SCRSTANDARD | D−6936 |
| OPC−4 | d5−JA | SCRSTANDARD | D−6936 |
| JA | d5−JA | SCRSTANDARD | D−6936 |

| | | | |
|---|---|---|---|
| JA−ILE | d5−JA | SCRSTANDARD | D−6936 |
| MEJA | d5−MEJA | IsoReag | IR−22054 |
| OPDA | d5−JA | SCRSTANDARD | D−6936 |
| 12−OH−JA | d5−JA | SCRSTANDARD | D−6936 |
| SA | d4−SA | isoreag | IR−30219−0.1 g |
| SAG | d4−SA | isoreag | IR−30219−0.1 g |
| Phe | N−(3−Indolylacetyl)−L−phenylalanine | Bide | BD9025−1 g |
| t−CA | trans−Cinnamic acid | Ron | R014152−5 g |
| 2−Coumarate | 2−Coumarate | TCI | C0394−5 G |
| MeSAG | 2−Methoxycarbonylphenyl beta−D−glucopyranoside | Heya | B017320 |

## Supplementary Table S2 Primer sequence for qRT−PCR

| Gene name | Gene Locus ID | Primer−F | Primer−R |
|---|---|---|---|
| *Csβ−actin* | HD.01G0021100 | TGAGGATATTCAGCCCCTTG | GCCTGGGTCGACCTACAATA |
| *CsANS* | HD.09G0021230 | CTTCAAGGGTATGGGAGCAA | CGTACTCAACTGTCGCAGGA |
| *CsSCPL* | HD.1307844 | ACCAGGCTTTGATGGTGAAC | CATTGAAAGCAGAGCAACCA |
| *CsDFR* | HD.06G0036240 | CACAGTTCGCGATCCTGATA | ACGTGGAAGACACCATGACA |
| *CsTCP* | HD.02G0007530 | TGCGAGATGTGAGATTCAGG | AAATCAGGAGGAGCAGCAAA |
| *CsMYB−related* | HD.03G0018090 | GCAGCTACATTCTGGCCTTC | AGTGCACACTGGCAACAGTC |
| *CsTIR1* | HD.01014854 | ATAGCCCTTTTGGTGACGTG | TGGCATCTTCTTTGCAAGTG |

## Supplementary Table S3 Design of AsODNs sequences for *CsARF2*, *CsMYC2*, and *CsMYB80*

| Gene | Primer−F | Primer−R |
|---|---|---|
| *CsARF2* (HD.14G0019970) −AsODNs | AGACAGTTGGAGGTCTTGCAAGGT | ACCTTGCAAGACCTCCAACTGTCT |
| *CsMYC2* (HD.07G0018360)−AsODNs | AAACTGCAACGGAATCCAGT | ACTGGATTCCGTTGCAGTTT |
| *CsMYB80* (HD.08G0030520) −AsODNs | TACATTGCCCAACACGGAAC | GTTCCGTGTTGGGCAATGTA |
| *CsARF2* (HD.14G0019970)−qRT−PCR | GATTGGAGAATCAGCCGCACTT | GGAAGAGCAGGACAGACACTATGA |
| *CsMYC2* (HD.07G0018360)−qRT−PCR | GCTTGTGAAGACTCCACCAGTG | TCTTACCTCGTCAGCGTCCATC |
| *CsMYB80* (HD.08G0030520) − qRT−PCR | CGGTTACCCACAAGCCCTTCTC | TGCTGGTCTCCTGCCGTGTA |
| *CsSCPL16* (HD.1307844)−qRT−PCR | GCTCCTCTTCCTCCTAGTGCTCTT | GGCGTACCTCCCTCATACTCTGTT |

## Supplementary Table S4 Contents of endogenous phytohormone components in tea shoots of different treatment groups (ng·$g^{-1}$ FW)

| Hormone category | Component | Ct−1 | Ct−2 | Ct−3 | PAC−1 | PAC−2 | PAC−3 | LS−1 | LS−2 | LS−3 | P−L−1 | P−L−2 | P−L−3 |
|---|---|---|---|---|---|---|---|---|---|---|---|---|---|
| GAs | GA5 | 8.56 | 8.86 | 8.71 | 4.28 | 4.69 | 3.81 | 7.04 | 8.34 | 8.05 | 5.41 | 5.20 | 5.00 |
| | GA3 | 9.26 | 8.61 | 9.17 | 10.45 | 9.46 | 9.07 | 7.92 | 9.25 | 7.95 | 5.36 | 5.28 | 5.19 |
| | GA4 | 2.59 | 2.37 | 1.97 | 1.58 | 2.07 | 2.10 | 0.00 | 0.00 | 0.00 | 2.08 | 1.23 | 2.30 |
| | GA8 | 0.72 | 0.50 | 0.99 | 1.21 | 0.76 | 0.40 | 0.77 | 1.00 | 0.91 | 0.62 | 0.77 | 0.58 |
| | GA9 | 1.20 | 1.38 | 1.18 | 1.13 | 1.20 | 1.50 | 1.48 | 1.32 | 0.64 | 0.00 | 0.00 | 0.00 |
| | GA7 | 0.25 | 0.11 | 0.12 | 0.05 | 0.07 | 0.07 | 0.07 | 0.07 | 0.06 | 0.00 | 0.00 | 0.00 |
| Auxins | TRA | 6.64 | 6.70 | 6.05 | 7.29 | 6.90 | 6.72 | 6.23 | 6.67 | 7.28 | 7.74 | 7.29 | 6.89 |
| | TRP | 24918.61 | 23050.30 | 25574.56 | 30019.14 | 29476.25 | 30562.05 | 28693.38 | 28511.14 | 28328.73 | 25367.89 | 24645.25 | 26090.54 |

| | | | | | | | | | | | | | |
|---|---|---|---|---|---|---|---|---|---|---|---|---|---|
| | IAA−Phe | 0.00 | 0.00 | 0.00 | 0.00 | 0.00 | 0.00 | 0.00 | 0.00 | 0.00 | 0.08 | 0.05 | 0.05 |
| | OxIAA | 18.03 | 21.50 | 19.76 | 33.16 | 34.06 | 32.27 | 27.05 | 26.03 | 26.54 | 22.85 | 21.05 | 22.06 |
| | IAA−Val−Me | 0.00 | 0.00 | 0.00 | 0.00 | 0.00 | 0.00 | 0.36 | 0.20 | 0.35 | 0.18 | 0.08 | 0.11 |
| | MEIAA | 0.34 | 0.38 | 0.44 | 0.26 | 0.38 | 0.34 | 0.20 | 0.30 | 0.36 | 0.33 | 0.37 | 0.33 |
| | IAM | 8.79 | 6.42 | 7.61 | 8.23 | 8.07 | 7.27 | 7.40 | 8.61 | 9.83 | 10.54 | 10.43 | 10.58 |
| | IAA−Ala | 0.71 | 0.61 | 0.54 | 0.82 | 0.90 | 1.13 | 0.87 | 0.67 | 0.66 | 1.17 | 0.69 | 1.31 |
| | IAA | 61.04 | 61.68 | 62.31 | 57.91 | 57.15 | 56.38 | 44.69 | 46.12 | 45.80 | 61.72 | 60.56 | 61.14 |
| | IAN | 0.18 | 0.00 | 0.00 | 0.44 | 0.50 | 0.69 | 0.40 | 0.21 | 0.30 | 0.25 | 0.41 | 0.51 |
| | IAA−Glc | 4.39 | 4.99 | 5.73 | 4.71 | 5.42 | 4.76 | 3.14 | 3.98 | 4.07 | 5.29 | 4.51 | 4.39 |
| | ICAld | 4.92 | 5.62 | 4.42 | 4.58 | 5.84 | 6.46 | 5.51 | 6.49 | 5.99 | 4.14 | 4.13 | 5.28 |
| | ICA | 2.79 | 2.68 | 2.07 | 2.14 | 2.25 | 3.35 | 2.95 | 2.56 | 2.97 | 2.82 | 1.69 | 1.78 |
| | Indole | 6714.46 | 6883.42 | 6545.51 | 9152.95 | 9019.08 | 9286.83 | 9015.21 | 9283.11 | 8958.34 | 6820.87 | 6849.70 | 6878.53 |
| | IAA−Asp | 2.54 | 2.67 | 2.68 | 2.94 | 3.70 | 3.86 | 3.01 | 3.20 | 2.61 | 4.26 | 4.42 | 4.15 |
| | BAP9G | 0.35 | 0.21 | 0.17 | 0.00 | 0.00 | 0.00 | 0.00 | 0.00 | 0.00 | 0.00 | 0.00 | 0.00 |
| | mT | 0.11 | 0.09 | 0.07 | 0.00 | 0.00 | 0.00 | 0.00 | 0.00 | 0.00 | 0.00 | 0.00 | 0.00 |
| | 2MeSiP | 0.00 | 0.00 | 0.00 | 0.07 | 0.06 | 0.06 | 0.00 | 0.00 | 0.00 | 0.00 | 0.00 | 0.00 |
| | K | 0.10 | 0.08 | 0.08 | 0.10 | 0.08 | 0.09 | 0.00 | 0.00 | 0.00 | 0.10 | 0.10 | 0.08 |
| | 2MeScZR | 1.38 | 1.37 | 1.80 | 1.70 | 1.51 | 1.36 | 1.38 | 1.54 | 1.70 | 1.55 | 1.47 | 1.30 |
| | DHZROG | 1.72 | 1.72 | 1.94 | 2.24 | 2.14 | 2.08 | 1.90 | 1.83 | 2.06 | 1.87 | 1.94 | 1.89 |
| | IP | 0.14 | 0.17 | 0.17 | 0.20 | 0.21 | 0.23 | 0.19 | 0.21 | 0.26 | 0.16 | 0.07 | 0.10 |
| **CTKs** | IPR | 0.31 | 0.24 | 0.26 | 0.35 | 0.31 | 0.34 | 0.34 | 0.28 | 0.33 | 0.18 | 0.20 | 0.22 |
| | iP7G | 0.22 | 0.29 | 0.32 | 0.53 | 0.24 | 0.34 | 0.26 | 0.29 | 0.29 | 0.21 | 0.22 | 0.18 |
| | tZ | 0.39 | 0.42 | 0.35 | 0.39 | 0.47 | 0.48 | 0.41 | 0.39 | 0.38 | 0.41 | 0.39 | 0.43 |
| | DHZ7G | 0.06 | 0.06 | 0.06 | 0.08 | 0.08 | 0.08 | 0.07 | 0.05 | 0.07 | 0.08 | 0.07 | 0.06 |
| | mT9G | 0.36 | 0.39 | 0.43 | 0.30 | 0.47 | 0.37 | 0.36 | 0.37 | 0.59 | 0.33 | 0.39 | 0.39 |
| | tZOG | 4.43 | 4.62 | 4.94 | 4.79 | 4.36 | 4.58 | 3.73 | 3.56 | 3.87 | 3.30 | 3.15 | 3.17 |
| | tZR | 1.93 | 2.38 | 2.32 | 3.32 | 3.23 | 3.42 | 2.14 | 2.56 | 2.72 | 2.21 | 2.59 | 2.44 |
| | tZRMP | 17.34 | 17.29 | 25.81 | 37.46 | 24.77 | 25.76 | 20.76 | 22.85 | 23.91 | 28.24 | 31.38 | 28.00 |

| | | | | | | | | | | | | | |
|---|---|---|---|---|---|---|---|---|---|---|---|---|---|
| | tZ9G | 4.36 | 4.32 | 3.24 | 2.52 | 3.33 | 3.66 | 3.34 | 3.29 | 2.57 | 3.33 | 2.70 | 3.80 |
| **JAs** | JA−Val | 0.30 | 0.27 | 0.31 | 0.54 | 0.61 | 0.55 | 0.60 | 0.51 | 0.55 | 0.40 | 0.44 | 0.45 |
| | MEJA | 15.63 | 14.54 | 15.62 | 37.22 | 37.78 | 38.34 | 33.14 | 32.83 | 32.51 | 14.87 | 15.52 | 16.38 |
| **ABAs** | ABA | 569.49 | 529.20 | 549.35 | 437.99 | 458.38 | 417.61 | 403.94 | 416.71 | 429.48 | 459.39 | 458.56 | 539.64 |
| | ABA−GE | 540.11 | 552.70 | 527.53 | 0.00 | 0.00 | 0.00 | 0.00 | 0.00 | 0.00 | 0.00 | 0.00 | 0.00 |
| | ABA−ald | 370.68 | 395.16 | 382.92 | 354.13 | 361.99 | 346.27 | 303.85 | 346.13 | 324.99 | 273.23 | 200.69 | 236.96 |
| **SAs** | SAG | 5059.09 | 5399.40 | 6662.41 | 4912.65 | 4918.47 | 4906.84 | 4738.97 | 4697.12 | 4655.28 | 4083.48 | 4170.06 | 4256.63 |
| | SA | 184.60 | 186.17 | 183.02 | 366.67 | 376.27 | 393.63 | 189.37 | 194.82 | 192.10 | 270.50 | 295.06 | 264.68 |
| | MeSAG | 145.15 | 125.45 | 135.30 | 136.65 | 144.93 | 128.37 | 268.91 | 276.54 | 284.18 | 147.46 | 154.94 | 162.41 |
| | Phe | 7808.63 | 7410.61 | 7845.33 | 8656.41 | 8582.13 | 8730.68 | 9251.65 | 9528.00 | 9478.59 | 8117.71 | 8520.86 | 8319.29 |
| | t−CA | 76.22 | 75.10 | 77.35 | 75.06 | 76.03 | 77.00 | 87.46 | 87.42 | 87.38 | 90.69 | 96.45 | 115.14 |
| | 2−Coumarate | 0.00 | 0.00 | 0.00 | 260.68 | 261.19 | 260.17 | 267.72 | 270.31 | 269.02 | 307.85 | 315.83 | 311.84 |

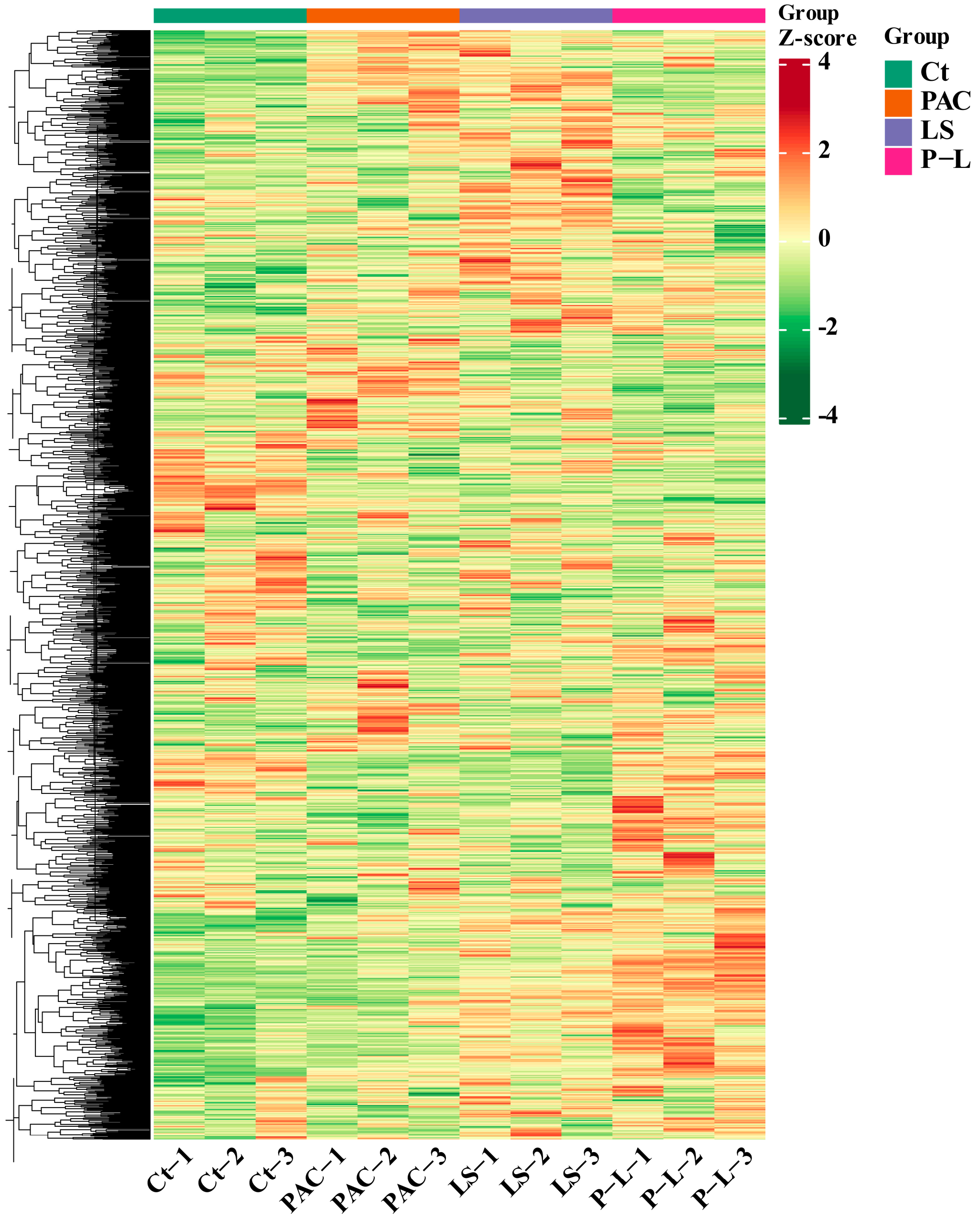


**Figure S1** Cluster heatmap of all detected metabolites in tea shoots under different treatments

## Supplementary Table S5 Quality evaluation of RNA−seq data

| Sample | Raw Reads | Clean Reads | Clean Base (G) | Error Rate (%) | Q20(%) | Q30(%) | GC Content (%) |
|---|---|---|---|---|---|---|---|
| CK−1 | 49,882,136 | 47,889,524 | 7.18 | 0.03 | 97.66 | 93.23 | 45.61 |
| CK−2 | 49,477,036 | 48,290,288 | 7.24 | 0.03 | 97.3 | 92.65 | 45.06 |
| CK−3 | 53,013,374 | 51,798,202 | 7.77 | 0.03 | 97.35 | 92.76 | 45.09 |
| PAC−1 | 51,895,474 | 50,643,510 | 7.6 | 0.03 | 97.42 | 92.92 | 45.13 |
| PAC−2 | 52,079,078 | 50,984,822 | 7.65 | 0.03 | 97.25 | 92.56 | 45.16 |
| PAC−3 | 47,254,870 | 46,304,574 | 6.95 | 0.03 | 97.34 | 92.71 | 45.07 |
| LS−1 | 49,956,286 | 48,864,688 | 7.33 | 0.03 | 97.37 | 92.82 | 45.09 |
| LS−2 | 51,253,944 | 50,209,048 | 7.53 | 0.03 | 97.11 | 92.14 | 45.05 |
| LS−3 | 49,082,810 | 47,843,588 | 7.18 | 0.03 | 97.56 | 93.27 | 45.1 |
| P−L−1 | 51,690,576 | 50,395,926 | 7.56 | 0.03 | 97.53 | 93.21 | 44.96 |
| P−L−2 | 48,720,142 | 47,618,644 | 7.14 | 0.03 | 97.56 | 93.28 | 45.14 |
| P−L−3 | 52,745,060 | 51,601,080 | 7.74 | 0.03 | 97.59 | 93.34 | 45.17 |

Note: Raw reads: the number of reads of the original sequencing data; Clean reads: the number of filtered reads; Clean bases: the number of Clean reads multiplied by the length of reads; Error rate: average error rate of sequencing base; Q20, Q30: the percentage of bases with sequencing quality greater than 99% and 99.9% in total bases; GC content: the percentage of the sum of G and C bases of Clean reads in total bases. CK: contrast; PAC: 50μmol·$L^{-1}$PAC for sample treatment; LS: 100μmol·$L^{-1}$LS treatment sample; P−L: 50μmol·$L^{-1}$PAC +100μmol·$L^{-1}$LS for sample treatment.

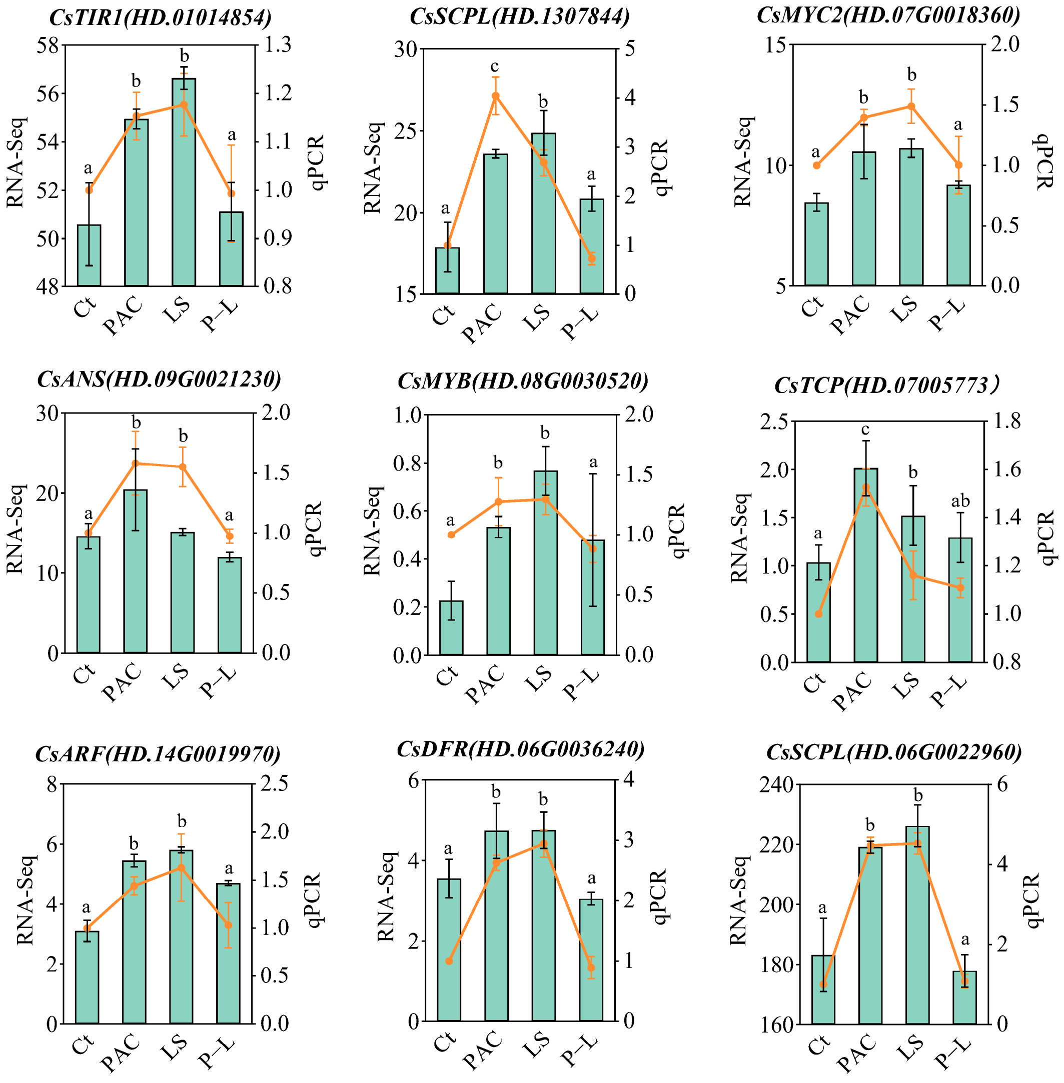


**Figure S2** FPKM value and qRT−PCR relative expression of differentially expressed genes. The histogram represents the FPKM value of the differentially expressed genes, and the line chart represents the relative expression of qRT−PCR of the differentially expressed genes. Different lowercase letters represent significant differences in gene expression detected by qRT−PCR at the $p<0.05$ level.

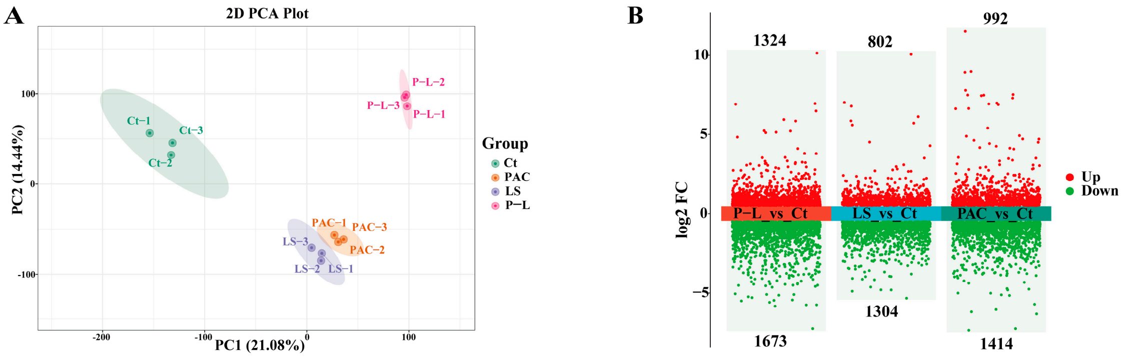


**Figure S3** Transcriptome analysis of tea shoots. (A) PCA diagram of the overall sample. (B) Number of differentially expressed genes.

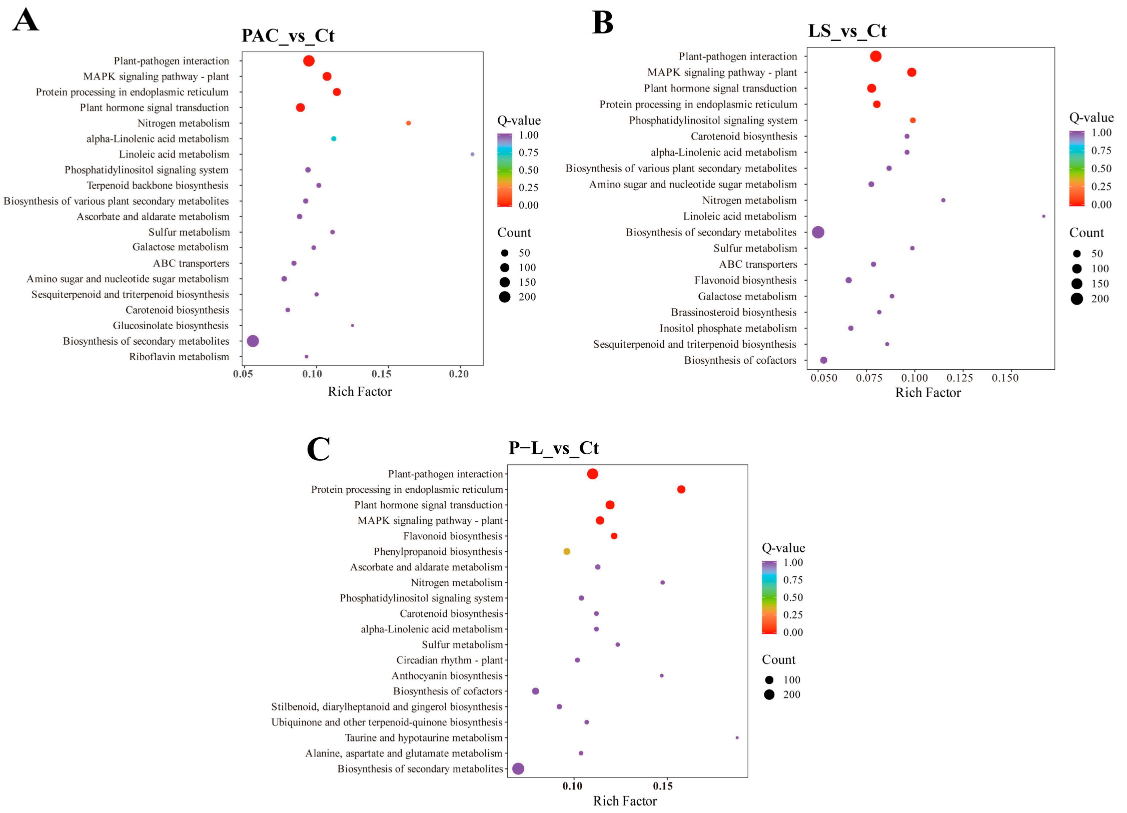


**Figure S4** KEGG enrichment bubble diagram of differential genes in different treatment groups. (A) Enrichment analysis of PAC vs Ct. (B) Enrichment analysis of LS vs Ct. (C) Enrichment analysis of P−L vs Ct.

## Supplementary Table S6 Transcription factor binding site of 4 structure genes

| Motif id | TF | Target | Start | Stop | Strand | Score | p−Value | q−Value | Matched sequence |
|---|---|---|---|---|---|---|---|---|---|
| MP00617 | *CsHB−WOX*(HD.01G0027770) | *CsSCPL*(HD.06G0022960) | 222 | 231 | + | 13.4894 | $1.20\times10^{-5}$ | 0.144 | aatcaatcat |
| MP00213 | *CsMYB*(HD.01G0029900) | *CsSCPL*(HD.06G0022960) | 1010 | 1020 | − | 13.0152 | $1.87\times10^{-5}$ | 0.0787 | GATTTAGTTAT |
| MP00238 | *CsZF−HD*(HD.01G0030230) | *CsSCPL*(HD.06G0022960) | 257 | 267 | − | 12.0597 | $6.22\times10^{-5}$ | 0.483 | ATATAATTAAT |
| MP00125 | *CsC2H2*(HD.02G0015370) | *CsDFR*(HD.06G0036240) | 1350 | 1369 | + | 11.0758 | $2.13\times10^{-5}$ | 0.12 | aaaaaaaccacaaaaaaaaa |
| MP00422 | *CsWRKY*(HD.02G0025310) | *CsSCPL*(HD.05G0027520) | 506 | 518 | + | 13.2121 | $1.73\times10^{-5}$ | 0.229 | aaaagtcaatttt |
| MP00556 | *CsERF*(HD.02G0032350) | *CsSCPL*(HD.06G0022960) | 1828 | 1842 | − | 10.8182 | $3.38\times10^{-5}$ | 0.179 | GTGGTGGTGGTGGTG |
| MP00408 | *CsWRKY*(HD.02G0032820) | *CsSCPL*(HD.05G0027520) | 506 | 518 | − | 10.7424 | $4.00\times10^{-5}$ | 0.577 | AAAATTGACTTTT |
| MP00302 | *CsAP2/ERF*(HD.02G0033890) | *CsSCPL*(HD.06G0022960) | 1829 | 1849 | − | 27.2 | $6.13\times10^{-11}$ | 9.71E−07 | GGTGGTGGTGGTGGTGGTGGT |
| MP00222 | *CsMYB*(HD.03G0004660) | *CsSCPL*(HD.06G0022960) | 405 | 417 | + | 12.5488 | $2.90\times10^{-5}$ | 0.361 | atactttccgtta |
| MP00320 | *CsHD−ZIP*(HD.03G0018290) | *CsSCPL*(HD.06G0022960) | 222 | 234 | − | 16.5909 | $1.16\times10^{-6}$ | 0.0136 | TAAATGATTGATT |
| MP00081 | *CsbHLH*(HD.03G0030020) | *CsSCPL*(HD.06G0022960) | 1540 | 1549 | − | 15.7727 | $1.36\times10^{-6}$ | 0.0216 | GGCCACGTGA |
| MP00570 | *CsC2C2*(HD.04G0020440) | *CsSCPL*(HD.1307844) | 1409 | 1422 | − | 16.2576 | $2.45\times10^{-7}$ | 0.00282 | AAAAAAGTAAAAAA |
| MP00100 | *CsbHLH*(HD.05G0001220) | *CsSCPL*(HD.06G0022960) | 1701 | 1708 | + | 12.7865 | $4.39\times10^{-5}$ | 0.546 | ccacgtgc |
| MP00090 | *CsSBP*(HD.05G0006190) | CsDFR(HD.06G0036240) | 1280 | 1295 | + | 13.4045 | $1.44\times10^{-5}$ | 0.133 | aattttgtacaataaa |
| MP00179 | *CsNAC*(HD.06G0029710) | *CsSCPL*(HD.05G0027520) | 1897 | 1915 | − | 5.07576 | $9.80\times10^{-5}$ | 1 | TTTATACTTGCAGCTGACG |
| MP00064 | *CsTCP*(HD.07005773) | *CsSCPL*(HD.06G0022960) | 1430 | 1439 | − | 13.0204 | $1.22\times10^{-5}$ | 0.194 | TGGGGTCCAC |
| MP00171 | *CsWRKY*(HD.07G0003420) | *CsSCPL*(HD.05G0027520) | 506 | 518 | + | 10.7424 | $5.73\times10^{-5}$ | 0.689 | aaaagtcaatttt |
| MP00248 | *CsBES1*(HD.07G0006010) | *CsSCPL*(HD.06G0022960) | 1699 | 1709 | − | 6.27273 | $7.75\times10^{-5}$ | 0.742 | GGCACGTGGAT |

| | | | | | | | | | |
|---|---|---|---|---|---|---|---|---|---|
| MP00084 | *CsbHLH*(HD.07G0018840) | *CsSCPL*(HD.06G0022960) | 1701 | 1708 | + | 11.7349 | $5.38\times10^{-5}$ | 0.414 | ccacgtgc |
| MP00334 | *CsMYB*(HD.08G0000370) | CsDFR(HD.06G0036240) | 870 | 884 | + | 12.2879 | $4.03\times10^{-5}$ | 0.569 | ttggactgattaggt |
| MP00296 | *CsMYB*(HD.08G0016970) | *CsSCPL*(HD.06G0022960) | 314 | 334 | + | 17.0366 | $9.82\times10^{-7}$ | 0.00964 | aaagatgaggataaaattgtg |
| MP00564 | *CsMYB*((HD.08G0030520) | *CsSCPL*(HD.1307844) | 894 | 908 | − | 13.3939 | $1.58\times10^{-5}$ | 0.168 | TTTAATTAGTTAAAC |
| MP00174 | *CsGARP−G2*(HD.09G0017610) | *CsSCPL*(HD.06G0022960) | 860 | 874 | + | 11.8571 | $4.88\times10^{-5}$ | 0.348 | cgattaatattctct |
| MP00548 | *CsAP2/ERF*(HD.09G0018910) | *CsSCPL*(HD.06G0022960) | 1830 | 1850 | + | 17.5455 | $6.69\times10^{-7}$ | 0.0106 | ccaccaccaccaccaccacca |
| MP00448 | *CsWRKY*(HD.09G0022600) | *CsSCPL*(HD.05G0027520) | 723 | 733 | − | 12.8939 | $2.68\times10^{-5}$ | 0.235 | ATAAGTCAAAT |
| MP00162 | *CsC2C2*(HD.10G0006870) | *CsSCPL*(HD.05G0027520) | 1561 | 1571 | − | 12.6418 | $8.20\times10^{-6}$ | 0.107 | CACTGCCACTG |
| MP00121 | CsNAC(HD.10G0014670) | CsDFR(HD.06G0036240) | 1709 | 1730 | − | 14.2273 | $4.18\times10^{-6}$ | 0.0579 | TACGTGTTCGGATCGTCA AATT |
| MP00139 | *CsAP2/ERF*(HD.11G0005680) | CsDFR(HD.06G0036240) | 966 | 982 | − | 13.4394 | $1.28\times10^{-5}$ | 0.135 | AAGGTGAGTTATGTTGA |
| MP00160 | *CsGARP−G2*(HD.11G0005710) | *CsSCPL*(HD.06G0022960) | 862 | 872 | − | 11.3939 | $9.28\times10^{-5}$ | 1 | AGAATATTAAT |
| MP00299 | *CsWRKY*(HD.11G0020630) | *CsSCPL*(HD.06G0022960) | 1795 | 1805 | + | 12.1667 | $4.54\times10^{-5}$ | 0.328 | ataagtcaaaa |
| MP00306 | *CsC2C2*(HD.11G0025110) | *CsSCPL*(HD.05G0027520) | 37 | 43 | − | 13.2632 | $9.66\times10^{-5}$ | 0.114 | TTGAAAA |
| MP00464 | *CsWRKY*(HD.12G0001660) | *CsSCPL*(HD.05G0027520) | 505 | 518 | − | 12.0455 | $2.86\times10^{-5}$ | 0.325 | AAAATTGACTTTTT |
| MP00439 | *CsNAC*(HD.12G0010900) | *CsSCPL*(HD.05G0027520) | 1897 | 1911 | − | 13.9091 | $1.14\times10^{-5}$ | 0.168 | TACTTGCAGCTGACG |
| MP00531 | *CsWRKY*(HD.1400400) | *CsSCPL*(HD.05G0027520) | 504 | 516 | − | 15.697 | $3.70\times10^{-6}$ | 0.0487 | AATTGACTTTTTT |
| MP00280 | *CsNAC*(HD.14G0005980) | CsDFR(HD.06G0036240) | 1839 | 1857 | − | 10.803 | $5.57\times10^{-5}$ | 0.879 | ATTTCTTACTTGTTAGGTT |
| MP00014 | *CsGARP−G2*(HD.14G0011470) | *CsSCPL*(HD.1307844) | 70 | 83 | + | 11.0161 | $5.70\times10^{-5}$ | 0.365 | gtaagatttgatgt |
| MP00099 | *CsbHLH*(HD.14G0017810) | *CsSCPL*(HD.06G0022960) | 1701 | 1708 | + | 11.9355 | $5.38\times10^{-5}$ | 0.429 | ccacgtgc |
| MP00251 | *CsWRKY*(HD.15G0007590) | *CsSCPL*(HD.05G0027520) | 504 | 518 | − | 13.4697 | $1.71\times10^{-5}$ | 0.239 | AAAATTGACTTTTTT |
| MP00232 | *CsHD−ZIP*(HD.15G0019350) | *CsSCPL*(HD.06G0022960) | 255 | 265 | + | 13.8939 | $1.63\times10^{-5}$ | 0.166 | tcattaattat |

## Supplementary Table S7 Prediction of hormone responsive elements in transcription factors

| Gene | Hormone responsive elements | Elements Sequence | Elements Position | Base length of elements | DNA positive and negative strands | Function |
|---|---|---|---|---|---|---|
| *CsTCP19* (HD.07005773) | AuxRR−core | GGTCCAT | 377 | 7 | − | cis−acting regulatory element involved in Auxin responsiveness |
| *CsMYB80* (HD.08G0030520) | TGACG−motif | TGACG | 1458 | 5 | + | cis−acting regulatory element involved in the MeJA−responsiveness |
| *CsMYB80* (HD.08G0030520) | AuxRR−core | GGTCCAT | 1655 | 7 | + | cis−acting regulatory element involved in Auxin responsiveness |
| *CsMYB80* (HD.08G0030520) | CGTCA−motif | CGTCA | 1458 | 5 | − | cis−acting regulatory element involved in the MeJA−responsiveness |
